# Glutamate Receptor-like (GLR) Family in *Brassica napus*: Genome-Wide Identification and Functional Analysis in Resistance to *Sclerotinia sclerotiorum*

**DOI:** 10.3390/ijms25115670

**Published:** 2024-05-23

**Authors:** Rana Muhammad Amir Gulzar, Chun-Xiu Ren, Xi Fang, You-Ping Xu, Mumtaz Ali Saand, Xin-Zhong Cai

**Affiliations:** 1 Key Laboratory of Biology and Ecological Control of Crop Pathogens and Insects of Zhejiang Province, Institute of Biotechnology, College of Agriculture and Biotechnology, Zhejiang University, Hangzhou 310058, China; 2 Centre of Analysis and Measurement, Zhejiang University, 866 Yu Hang Tang Road, Hangzhou 310058, China; 3 Department of Botany, Shah Abdul Latif University, Khairpur 66020, Sindh, Pakistan; 4 Hainan Institute, Zhejiang University, Sanya 572025, China

**Keywords:** bioinformatics, *Brassica napus*, glutamate receptor, resistance, *Sclerotinia sclerotiorum*

## Abstract

Plant glutamate receptor-like channels (GLRs) are homologs of animal ionotropic glutamate receptors. GLRs are critical in various plant biological functions, yet their genomic features and functions in disease resistance remain largely unknown in many crop species. Here, we report the results on a thorough genome-wide study of the *GLR* family in oilseed rape (*Brassica napus*) and their role in resistance to the fungal pathogen *Sclerotinia sclerotiorum*. A total of 61 *GLRs* were identified in oilseed rape. They comprised three groups, as in *Arabidopsis thaliana*. Detailed computational analyses, including prediction of domain and motifs, cellular localization, *cis*-acting elements, PTM sites, and amino acid ligands and their binding pockets in BnGLR proteins, unveiled a set of group-specific characteristics of the BnGLR family, which included chromosomal distribution, motif composition, intron number and size, and methylation sites. Functional dissection employing virus-induced gene silencing of *BnGLRs* in oilseed rape and *Arabidopsis* mutants of *BnGLR* homologs demonstrated that *BnGLR35*/*AtGLR2.5* positively, while *BnGLR12*/*AtGLR1.2* and *BnGLR53*/*AtGLR3.2* negatively, regulated plant resistance to *S. sclerotiorum*, indicating that *GLR* genes were differentially involved in this resistance. Our findings reveal the complex involvement of *GLRs* in *B. napus* resistance to *S. sclerotiorum* and provide clues for further functional characterization of *BnGLRs*.

## 1. Introduction

Calcium is a second messenger that plays essential roles in a variety of plant processes, including growth, development, and stress responses [1]. Calcium channels are pivotal to controlling calcium homeostasis across the plasma membrane [2]. Glutamate receptor-like channels (GLRs), which are plant homologs of mammalian ionotropic glutamate receptors, are amino acid-sensing calcium channels [3,4]. The plant *GLR* gene family was first reported in *A. thaliana*, whose total 20 gene members were clustered into 3 major groups: *AtGLR-I*, *AtGLR-II*, and *AtGLR-III* [5]. Later, *GLR* gene families were identified in other plant species such as sugarcane [6], bean [7], rice [5], tomato [8], Rosaceae species [9], banana [10], cabbage [11], radish [12], soybean [13], and *Brassica* species [14]. Nevertheless, comprehensive characterization of motifs, PTM sites, and amino acid ligands and their binding pockets in GLR proteins has not yet been conducted.

Generally, plant GLRs contain an extracellular amino-terminal domain (ATD), a ligand-binding domain (LBD) comprising segments S1 and S2, a transmembrane domain consisting of four membrane helices (M1 to M4), and a cytoplasmic tail (CTD), arranged in the order ATD-S1-M1-M2-M3-S2-M4-CTD [15]. Structural analyses reveal that the amino acid ligands interacting with AtGLRs include alanine (Ala), asparagine (Asn), cysteine (Cys), glutamic acid (Glu), glycine (Gly), methionine (Met), and serine (Ser) [16]. Binding of the ligand amino acids to GLRs leads to calcium influx, which consequently regulates plant biological processes [17].

GLRs are involved in a variety of plant processes including growth, development, and resistance/tolerance to biotic and abiotic stresses [18]. For example, *Arabidopsis AtGLR1.1*, *AtGLR3.1*, and *AtGLR3.5*; moss (*Physcomitrium patens*) *PpGLR1*; and rice *OsGLR3.4* are involved in growth and development [17,19,20,21]. *AtGLR1.2*, *AtGLR1.3*, and *AtGLR3.4* are related to tolerance to abiotic stresses such as cold [22] and salt [23], whereas *AtGLR3.3* and a small radish *GLR* play important roles in plant disease resistance [12,24,25,26]. As an example, *AtGLR3.3* is essential to plant resistance against diverse pathogens including *Hyaloperonospora arabidopsidis* [24], *Botrytis cinerea* [25], and *Pseudomonas syringae* pv *tomato* DC3000 [26] via activating salicylic acid-dependent defenses. Collectively, GLRs are multi-functional and their roles in plant biological processes are likely gene member-dependent [27]. Additionally, to date, results regarding GLRs are mainly obtained in the model plant species. Functional studies of the GLR family in disease resistance in crop species such as horticultural crops are scarce.

Oilseed rape (*Brassica napus*) is one of the most important oil crops, yet the functions of *GLRs* in oilseed rape disease resistance remain unclear. In this study, we performed comprehensive computational and functional analyses of *BnGLRs* to clarify their genome-wide constitution and sequence characteristics and their role in plant disease resistance against *Sclerotinia sclerotiorum*, one of the most important pathogens in oilseed rape. In total, 61 *GLR* genes were identified from the genome of the oilseed rape cultivar ZS11. Systematical analyses of the phylogeny, chromosomal distribution, domains and motifs, promoter *cis*-acting elements, pore morphology, post-translational modification (PTM) sites, and amino acid ligands for BnGLRs demonstrated family-conserved and group-specific characteristics of the BnGLR family. Virus-induced gene silencing (VIGS) analyses in oilseed rape and homolog gene mutant analyses in *Arabidopsis* revealed that *BnGLR35* positively, while *BnGLR12* and *BnGLR53* negatively, modulated plant resistance to *S. sclerotiorum*. Our findings demonstrate the functional complexity of the *GLR* gene family in plant disease resistance.

## 2. Results

### 2.1. Identification of GLR Family in Oilseed Rape

The 20 AtGLR protein sequences were collected from The Arabidopsis Information Resource (TAIR) to search against the *B. napus* genome via BLASTp in the Phytozome database. A total of 61 BnGLR candidate protein sequences were retrieved from the *B. napus* genome (Chinese cultivar ZS11). The BnGLR proteins were generally basic or near-neutral proteins, with an average PI (iso-electric point) value of 7.74 ranging from 5.89 (BnGLR2) to 9.59 (BnGLR61). They were plasma membrane proteins with an average size of 878.4 amino acids (aa) ranging from 585 aa (BnGLR23) to 1068 aa (BnGLR59) (Table 1), which corresponded to an average molecular weight (MW) of 93,319.28 Da ranging from 66,023 Da (BnGLR23) to 120,615.57 Da (BnGLR59). Subcellular localization analyses using the CELLO webtool predicted that all BnGLR proteins were localized in plasma membrane (Table 1).

The *BnGLR* genes contained 4–7 exons and dominantly 4–6 introns (Table 1). The chromosomal distribution of *BnGLRs* was group-specific. *BnGLR* genes from Group I (*BnGLR1*–*BnGLR19*) were distributed in A01 (*BnGLR5*), A02 (*BnGLR16* and *BnGLR17*), A06 (*BnGLR12*), A09 (*BnGLR9*), A10 (*BnGLR2* and *BnGLR3*) and C01 (*BnGLR6* and *BnGLR7*), C02 (*BnGLR14*, *-15*, *-18* and *-19*), C07 (*BnGLR13*), and C09 (*BnGLR1*, *-8*, *-10*, and *-11*), while that of *BnGLR4* was unknown. *BnGLRs* from Group II (*BnGLR20* and *BnGLR36*) were distributed in A03 (*BnGLR20*), A04 (*BnGLR22*, *-23*, *-25*, *-26*, *-27*, and *-28*), A05 (*BnGLR24*) and C02 (*BnGLR36*), C03 (*BnGLR21*), C04 (*BnGLR29*, *-30*, *-31*, *-32*, and *-33*), and C09 (*BnGLR35*), while that of *BnGLR34* was unknown. *BnGLRs* from Group III (*BnGLR37–BnGLR61*) were distributed in A03 (*BnGLR57*), A07 (*BnGLR48* and *BnGLR50*), A08 (*BnGLR37*, *-38*, and -*39*), A09 (*BnGLR42* and *BnGLR56*), A10 (*BnGLR40* and *BnGLR45*) and C01 (*BnGLR52* and *BnGLR53*), C03 (*BnGLR47* and *BnGLR58*), C04 (*BnGLR43*, -*44*, -*60*, and *-61*), C05 (*BnGLR41*, *-46*, and *-49*), C07 (*BnGLR51*), C08 (*BnGLR54* and *BnGLR55*), and C09 (*BnGLR59*) (Table 1). Collectively, *BnGLRs* were distributed in all oilseed rape chromosomes of the A and C sub-genomes except C06. However, the group-wide chromosomal distribution of *BnGLRs* was uneven, with the absence of Group I *BnGLRs* in A03–A05, A07–A08, A10, C03–C06, and C08, Group II *BnGLRs* missing from A01–A02, A06–A10, C01, and C05–C08, and Group III *BnGLRs* lacking in A01–A02, A04–A06, C02, and C06.

### 2.2. Phylogeny of BnGLR and AtGLR Families

**Figure 1:**
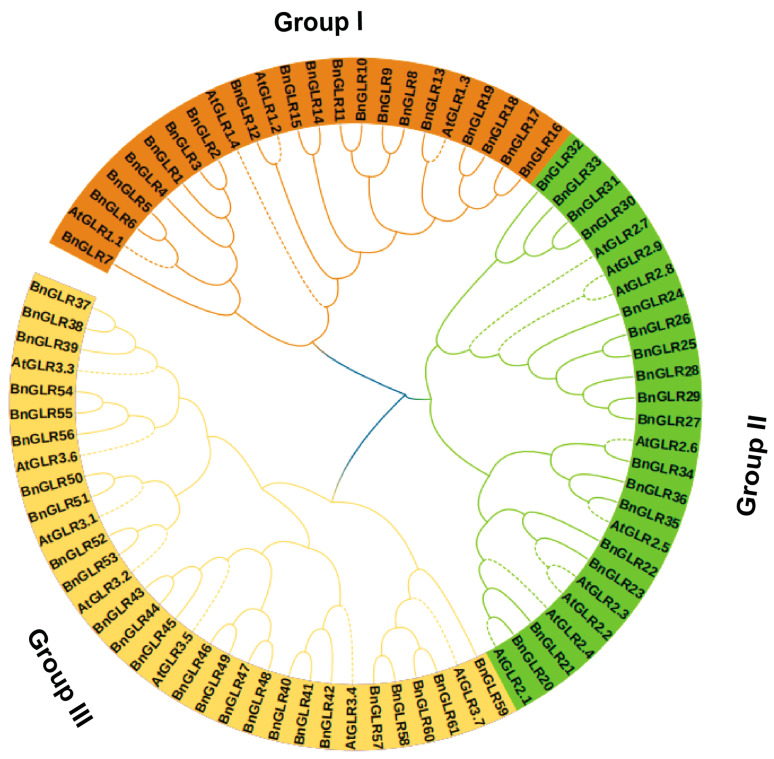
Phylogenic tree for AtGLR and BnGLR proteins. The maximum-likelihood (ML) phylogenetic tree of the AtGLR and BnGLR proteins was constructed by MEGA X software (v10.2.2) with 1000 bootstrap replicates. Dotted lines represent the AtGLRs, whereas plain lines stand for BnGLRs. Distinct colors indicate different groups of GLRs, Group I in brown, Group II in green, and Group III in yellow.

To elucidate the evolution of the BnGLR family, the maximum likelihood phylogenetic tree was generated for 20 AtGLR and 61 BnGLR proteins (Figure 1). Based on the phylogenetic tree, the BnGLR proteins were clearly clustered group-wide with AtGLRs into three major groups as Groups I, II, and III. The size and distribution of the three BnGLR groups were unequal to those of AtGLRs (Figure 1). Group I contained 19 BnGLRs (BnGLR1–BnGLR19) and 4 AtGLRs, Group II carried 17 BnGLRs (BnGLR20–BnGLR36) and 9 AtGLRs, while group III consisted of 25 BnGLRs (BnGLR37–BnGLR61) and 7 AtGLRs. This result demonstrated the significant and group-dependent expansion of BnGLRs in comparison with AtGLRs.

### 2.3. Conserved Motifs of BnGLR Proteins

**Figure 2:**
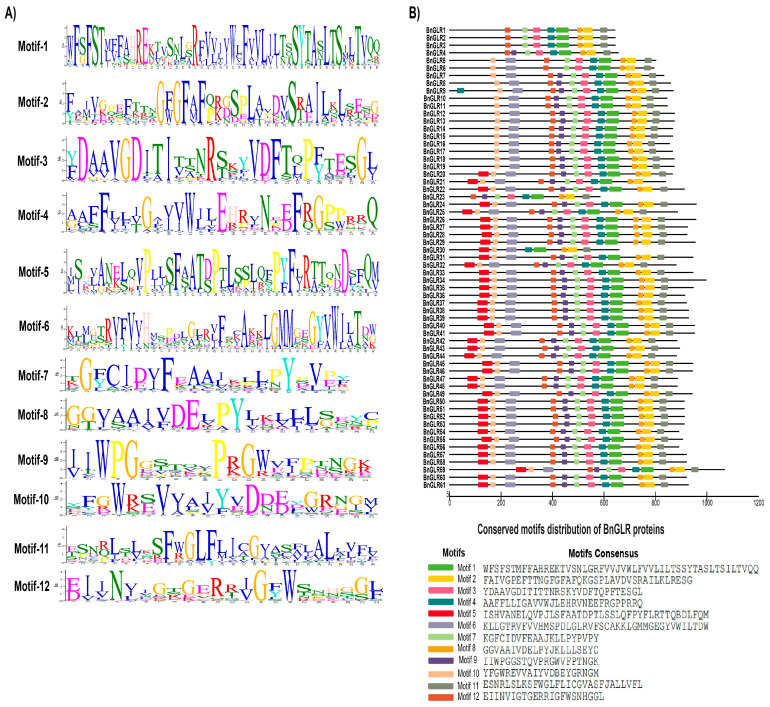
Conserved motifs among BnGLR proteins. (**A**) Logos of the predicted motifs. The BnGLR-specific motif logos were generated by using weblogo3 (v3.7). The bit scores for each position in the sequence are indicated to the left. (**B**) Motif profile of BnGLRs. The conserved motifs identified by MEME [28] are indicated by different colors. The consensus of each motif is provided.

To further obtain clues for the functions of BnGLRs, the conserved motifs of BnGLR proteins were predicted using MEME. Consequently, 12 motifs with 20–50 aa that were conserved in the BnGLR protein family were generated (Figure 2A). The structure of BnGLR proteins was group-dependent and dominated by a motif organization of 5-10-6-12-9-7-3-4-1-8-2-11 or lacking the N-terminal motif 5 (Figure 2B). Most of the proteins within the same group had common features in terms of motif composition and distribution. All BnGLR proteins of Groups II and III exhibited the motif pattern 5-10-6-12-9-7-3-4-1-8-2-11, except one protein (BnGLR23), which was shorter with a lack of the N-terminal triple motifs 5-10-6. BnGLR proteins of Group I dominated with the motif pattern 10-6-12-9-7-3-4-1-8-2-11, which lacked the N-terminal motif 5 and was otherwise identical to that of Groups II and III BnGLRs. The exceptions were BnGLR9, with additional N-terminal motif 4; BnGLR5 and BnGLR6, which lacked motif 9; BnGLR4, which lacked motifs 10 and 6; and BnGLR1-3, which lacked motifs 10, 6, and 9 (Figure 2B).

Intriguingly, multiple alignment of the ion channel domain by ClustalW revealed the existence of the conserved motif “SYTANLTS” in the C-terminus of motif 1 among BnGLRs, which was similar but not identical to the known functional motif “SYTANLAA” reported in two rat iGluRs and *Rosaceae* and *Arabidopsis* GLRs to be involved in ion exchange or transportation through the plasma membrane (Figure S1). Additionally, 12 amino acids, F (9), E (20), F (27, 42, 44), S (45), R (61), W (67), F (69), S (77), Y (78), and A (80), were identical in this domain of all BnGLRs (Figure S1).

### 2.4. Domains and Gene Structures of BnGLRs

Domain composition analyses revealed that all BnGLRs contained a ligand-binding domain and an ion channel domain, a bacterial periplasmic substrate-binding protein domain (PBPe) (accession: SM000079), or a low complexity region (LCR) and 2–4 short transmembrane TMDs in “SMART” database, while a periplasmic_binding_protein_type_1 (PBP1) and a PBP2, or, more specifically, a PBP1_GABAb_receptor_plant (accession: cd06366) and a Glur_Plant (accession: cd13686) in the NCBI-CDD database. Remarkably, BnGLR21 carried a B3 domain (Table 1; Figures S2 and S3). Whether it is functional in regulating the transcription of target genes remains to be experimentally confirmed.

**Figure 3:**
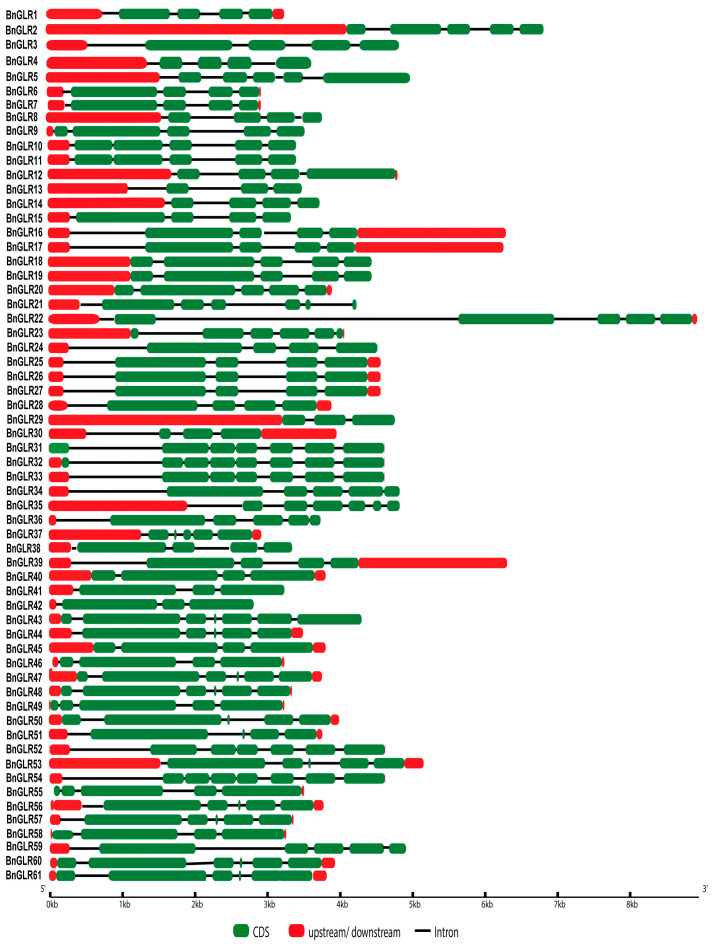
The exon–intron structures of 61 *BnGLR* genes. The constituents of *BnGLR* genes are indicated in differentially colored shapes. CDS: green box; upstream/downstream: red box; introns: black lines.

To study the *GLR* gene structure, the distribution of the exons and introns in *BnGLR* genes was identified. The number of introns ranged from 3 to 7. On average the introns were predicted to be 4.05, 4.47, and 5.52 in Group I, II, and III, respectively, indicating that the *BnGLR* genes of Group III contained more introns than those of the other groups (Table 1; Figure 3). Additionally, the size of introns also differed significantly in the *BnGLR* genes of different groups (Figure 3). Together, these results indicate that the gene structure of *BnGLR* genes is group-specific.

### 2.5. Cis-Acting Elements in Promoters of BnGLR Genes

**Figure 4:**
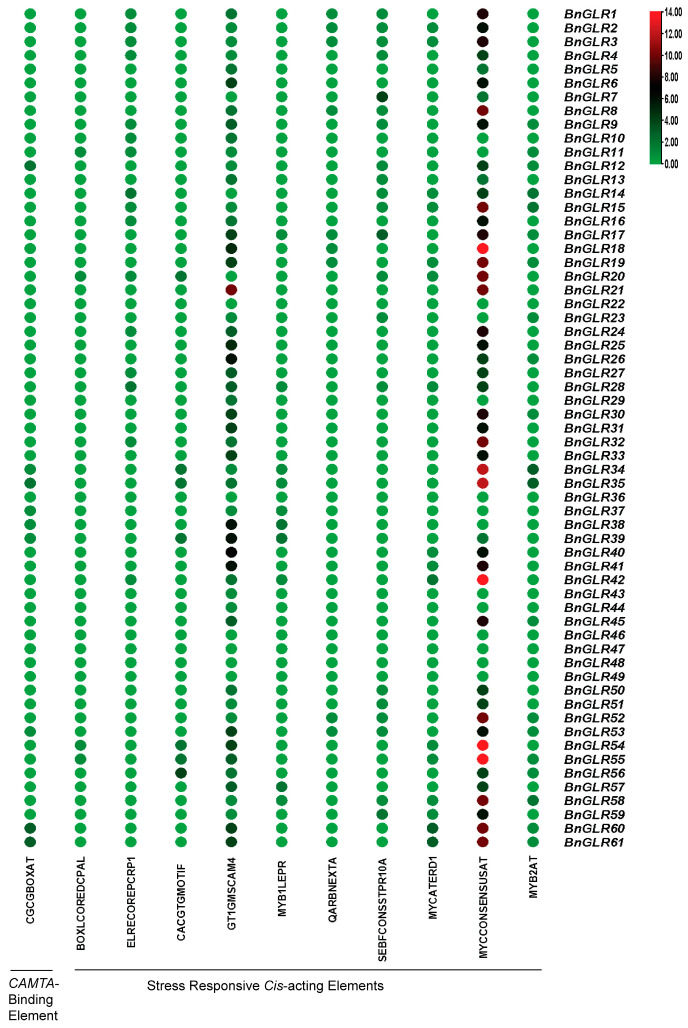
*Cis*-acting elements in *BnGLR* gene promoters. Major stress responsive *cis*-elements predicted in *BnGLRs* are shown. The types of *cis*-elements are provided. The bubble heat map showing the number of elements was prepared using the TB-tool (v2.084) [29].

In order to obtain preliminary clues for *BnGLR* gene function, *cis*-acting elements in the upstream (1.5 kb) sequence of *BnGLR* genes were analyzed using the search scan program in the PLACE database. Generally, very few (3.15%) CAMTA-binding *cis*-acting elements (CGCG-box) were predicted in *BnGLR* promoters (Figure 4). Only nine *BnGLRs*, one in Group I (*BnGLR12*), two in Group II (*BnGLR34* and *BnGLR35*), and six in Group III (*BnGLR37*, *-38*, *-39*, *-53*, *-60*, and *-61*), contained 1~2 CGCG-boxes, indicating that these genes might be regulated by CAMTA3. *BnGLRs* contained several biotic stress responsive elements such as BOXLCOREDCPAL, MYB1LEPR, and SEBFCONSSTPR10A (Figure 4). Two *BnGLRs*—*BnGLR21* and *BnGLR39*—from Group II contained the maximum (10) biotic-stress responsive elements in their promoter, whereas two *BnGLRs*—*BnGLR22* and *BnGLR36*—from the same group and four *BnGLRs* from Group III (*BnGLR46*, *-47*, *-48*, and *-49*) did not have any single biotic-stress related *cis*-elements. Moreover, *BnGLRs* carried a set of abiotic-stress responsive *cis*-elements such as ELRECOREPCRP1, GT1GMSCAM4, MYCATERD1, MYCONSENSUSAT, and MYB2AT (Figure 4). Two *BnGLRs* from Group III (*BnGLR42* and *BnGLR55*) contained the maximum number (16) of these *cis*-acting elements, while *BnGLR10* (Group I), *BnGLR22*, *-29*, and *-36* (Group II) and eight members from Group III (*BnGLR37*, *-38*, *-43*, *-44*, *-46*, *-47*, *-48*, and *-49*) did not have a single element. These results indicated that the *BnGLR* family might be widely involved in biotic- and abiotic-stress responses but in a member-dependent manner (Figure 4).

### 2.6. Tertiary Structure of BnGLR Proteins

**Figure 5:**
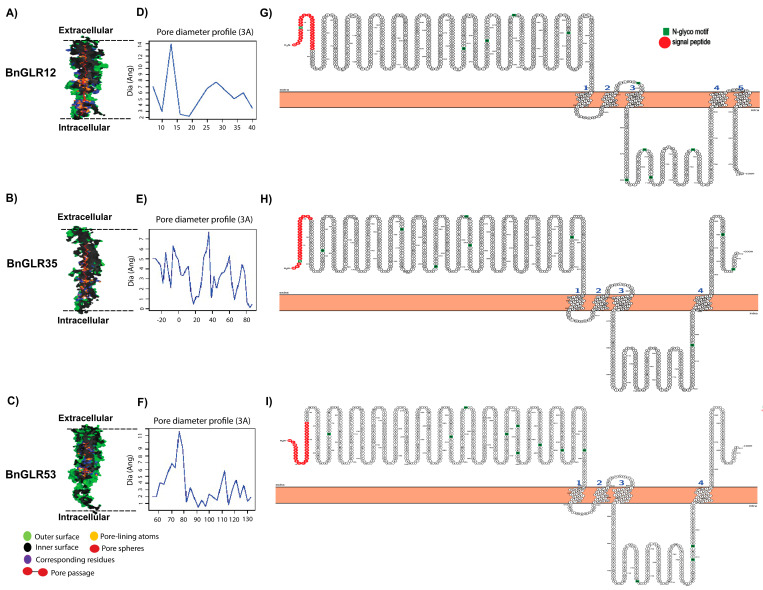
Pore morphology, dimensions, and topology of BnGLR proteins. (**A**–**C**) Protein tertiary structures of BnGLRs. Pore structure of three BnGLRs are shown; (**D**–**F**) pore diameter profile of BnGLRs. Pore dimensions were obtained using the PoreWalker software (v1.0); (**G**–**I**) topology of BnGLRs. Secondary structure of three BnGLRs was visualized by the online tool Protter (v1.0). The numbers count the transmembrane domains.

To examine whether BnGLRs were possible transmembrane protein channels, the PoreWalker webserver was used to predict the tertiary structures of three BnGLR proteins representing each group, BnGLR12, BnGLR35, and BnGLR53. The pore structure and 3D geometry of these BnGLR proteins were shown to fit with a pore morphology that longitudinally passed through the extracellular to intracellular opening of the proteins (Figure 5A–F). The pore size and constraints, which are considered as selective barriers, were predicted as shown in Figure 5A–F. Moreover, the Protter online tool was used for the structural visualization of these BnGLR proteins. The results showed that these BnGLR proteins were predicted to be membrane proteins with four transmembrane domains (Figure 5G–I). Together, these data support the BnGLR proteins to be transmembrane channels.

### 2.7. Amino Acid Ligands for BnGLRs

The interaction between 61 BnGLR proteins (as receptors) and 7 important amino acid ligands (Ala, Asn, Cys, Gln, Gly, Met, Ser) were observed based on ligand positions on receptor-binding pockets. Most BnGLR proteins had different binding sites for each of the above mentioned amino acid ligands but at distinct positions (Table S2). BnGLRs interacted with different amino acids via hydrogen bonding. BnGLR37 did not bind to any of the examined amino acids; BnGLR7 interacted uniquely with Ala; BnGLRs -3, -17, -30, -35, -36, and -39 interacted with two amino acids; and BnGLRs -2, -5, -8, -21, -33, and -51 bound to all seven amino acid ligands but at distinct sites. Distinguished from hydrogen bond interactions, all BnGLRs had hydrophobic interactions with all the amino acid ligands at different binding pockets consisting of 5–10 amino acids, most frequently Met, Phe, Thr, Arg, Leu, Ser, Glu, Tyr, Gly, and Ala. The most abundant binding residues in BnGLR receptor proteins through hydrogen bonds were Glu (867) in receptor BnGLR53 and Ala (76, 946, and 346) in BnGLR15, -25, and -60, respectively. Additionally, Arg (765) in BnGLR47, Tyr (432) in BnGLR52, and Asn (335 and 352) in BnGLR22 and BnGLR24 were also observed, respectively. On the contrary, binding residues through hydrophobic bonds were widely predicted in BnGLR proteins. They were Phe (335), Ala (273 and 344), Met (229), and Asp (175) in BnGLRs in Group I; Phe (333), Asp (260), and Arg (27) in BnGLRs in Group II; and Phe (339, 362), Asp (905), Ala (346), Leu (368), and Val (55, 79) in BnGLRs in Group III. Only five amino acid residues in BnGLRs bound to some of the above mentioned ligands through external bonds. These included Asp (175) and Ser (149) in BnGLR5, Gln (588) in BnGLR6, Glu (418) in BnGLR7, Pro (846) in BnGLR17, and Glu (732) in BnGLR33 (Table S2). These potential bindings need to be proved with further biochemical assays.

### 2.8. Post-Translational Modification Sites in BnGLRs

#### 2.8.1. Phosphorylation

**Figure 6:**
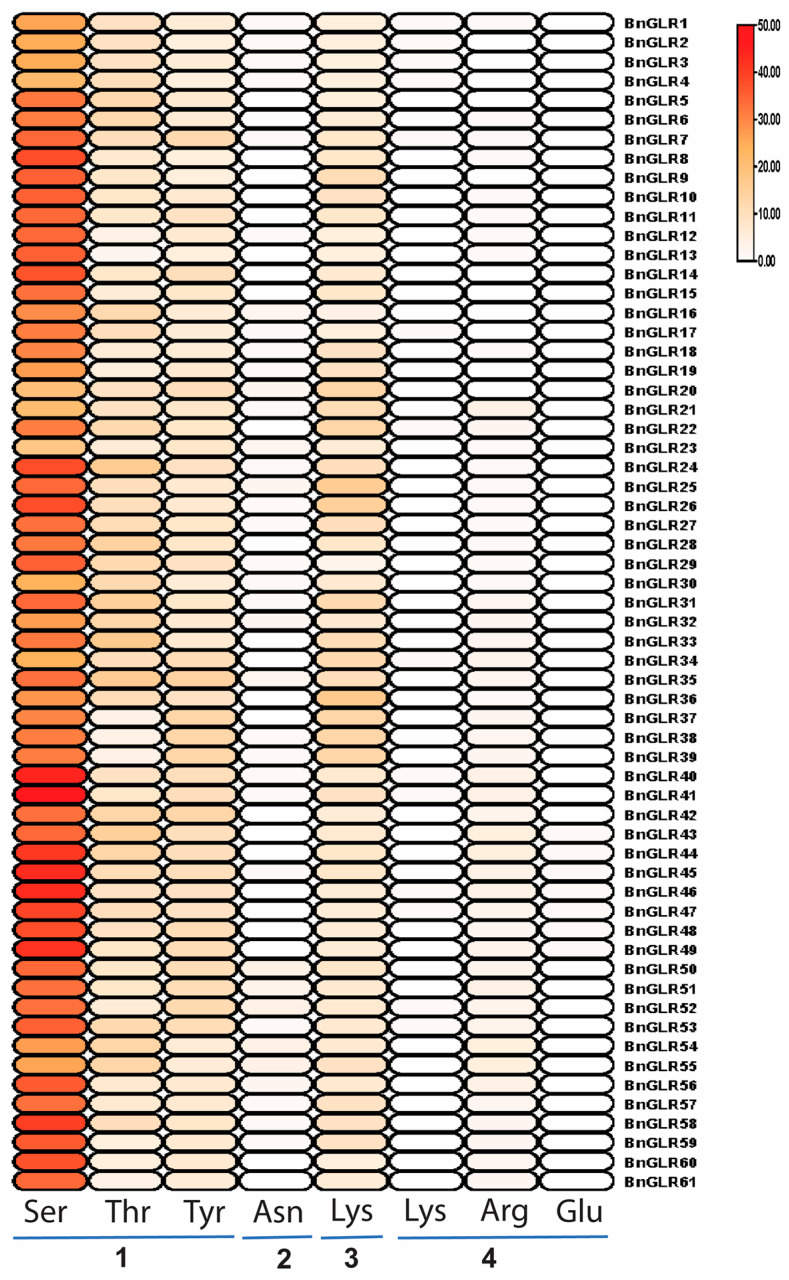
Predicted post-translational modification sites in 61 BnGLR proteins. TBtool was used to draw heatmap [29]. Bubble colors (white to red) indicate the number of PTM sites. 1. Phosphorylation sites (Ser—serine; Thr—threonine; Tyr—tyrosine). 2. N-Glycosylation site (Asn—asparagine). 3. Sumoylation site (Lys—lysine). 4. Methylation sites (Lys—lysine; Arg—arginine; Glu—glutamate).

The attachment of the phosphate group to the serine, threonine, and tyrosine amino acids of BnGLR proteins was predicted. Generally, all BnGLR proteins contained all these three phosphorylation sites. The maximum putative phosphorylation serine (48) and threonine (17) were found in BnGLR41 and BnGLR33, respectively. Whereas BnGLR37, -38, -39, and -42 had the maximum number (13) of putative phosphorylation tyrosine in their protein sequences (Figure 6, Table S3). This result indicated that BnGLRs are likely phosphorylation-inducible proteins.

#### 2.8.2. Glycosylation

There are four main categories of glycosylation based on the linkage between amino acids and sugars: N-linked glycans, O-linked glycans, GPI anchors, and C-mannosylation. Glycosylation analyses revealed the O-linked glycans in BnGLRs and AtGLRs, which were characterized by the interaction of a sugar with the hydroxyl group of serine or threonine. BnGLR50, BnGLR52, BnGLR54, and BnGLR55 carried the maximal four “Asn” residues, while 24 BnGLRs out of 61 did not contain any “Asn” residue (Figure 6, Table S3). This result implied that only some members of BnGLRs can be glycosylated.

#### 2.8.3. Sumoylation

Sumoylation sites were characterized by a covalent bond of small ubiquitin-like modifiers to a specific lysine residue through an enzymatic action. All BnGLRs had sumo sites. A maximum of 17 sumoylation sites were predicted in BnGLR36. The positions and scores of these sumoylation sites are clearly marked in Figure 6 and Table S3. This result suggests that all BnGLRs can likely be sumoylated.

#### 2.8.4. Methylation

The methylation sites in BnGLR proteins were predicted by using the online webserver, MASA. Methylation sites in lysine (Lys), arginine (Arg), and glutamate (Glu), but not in asparagine (N), were found in BnGLRs. The methylation site frequency was highest in Arg, followed by Lys, and lowest in Glu, which appeared only in six BnGLRs in Group III. Overall, the methylation sites in BnGLRs appeared to be site- and group-dependent. Group III BnGLRs contained more methylation sites, especially in Arg and Glu (Figure 6, Table S3).

Collectively, all BnGLRs carried phosphorylation and sumoylation sites, while glycosylation and methylation sites seemed to be BnGLR-dependent. Some Group I BnGLRs did not contain any methylation sites, while the absence of glycosylation site in BnGLRs was not group-dependent.

### 2.9. Response of BnGLRs to the Pathogen Sclerotinia sclerotiorum

**Figure 7:**
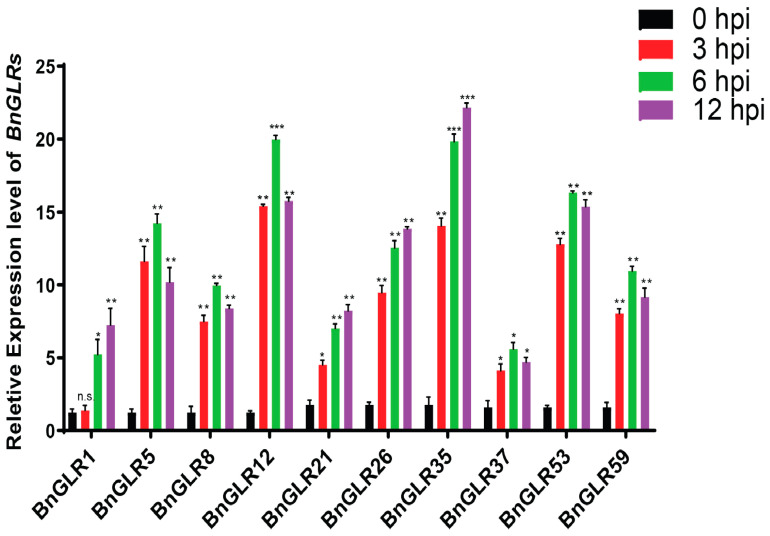
Transcriptional regulation of *BnGLRs* in response to *S. sclerotiorum* infection in oilseed rape plants. Gene expression was detected by qRT-PCR, in which *BnActin7* gene was used as reference. This experiment was performed with three biological replicates, each yielding similar results. The asterisks indicate significant differences (* *p* ≤ 0.05, ** *p* ≤ 0.01, *** *p* ≤ 0.001, n.s.—not significant) of *BnGLR* gene expression in four time points (0, 3, 6, 12 h post-inoculation (hpi)) when statistically analyzed by Student’s *t*-test.

To analyze the response of *BnGLR* genes to the necrotrophic pathogen *S. sclerotiorum* (*Ss*), expression analysis was performed using quantitative real-time PCR (qRT-PCR). The primers that were used for expression analysis are listed in Table S1. The *Ss* was inoculated on oilseed rape leaves and sampled at 3, 6, and 12 h post-inoculation (hpi) and mock-inoculated samples were collected as a control. A total of 10 *BnGLR* genes, including *BnGLR1*, *-5*, *-8*, and *-12* (Group I), *BnGLR21*, *-26*, and *-35* (Group II), and *BnGLR37*, *-53*, and *-59* (Group III) were selected, representing each group for expression analysis. Results showed that all genes were upregulated significantly in response to *Ss* inoculation. However, the extent of upregulation of *BnGLR* expression differed obviously. The expression was highest with a 23-fold peak enhancement at 12 hpi for *BnGLR35*, 20-fold at 6 hpi for *BnGLR12*, and 17-fold at 6hpi for *BnGLR53*, while it was lowest with a 5-fold peak enhancement at 6 hpi for *BnGLR37* and 7~8-fold at 12 hpi for *BnGLR1* and *BnGLR21* (Figure 7). This result indicated that the upregulation of *BnGLR* expression was not group-dependent. Generally, *BnGLRs* were induced remarkably in response to *Ss* inoculation, indicating that these *BnGLRs* might be involved in resistance to *Ss*.

### 2.10. Silencing of BnGLR12, BnGLR35, and BnGLR53 Distinctly Altered Oilseed Rape Resistance against S. sclerotiorum

**Figure 8:**
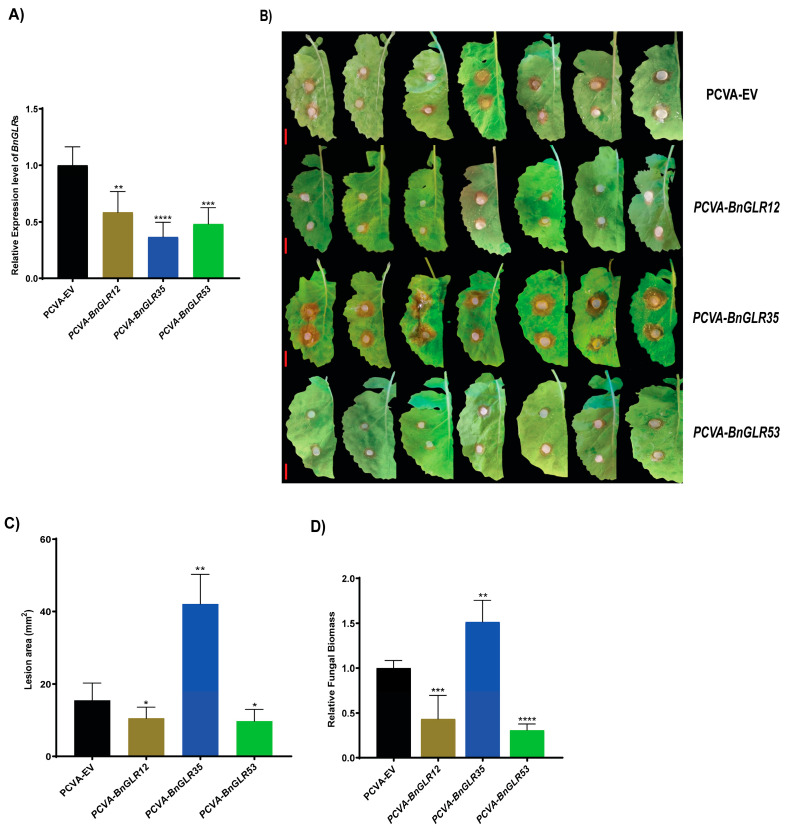
Virus-induced gene silencing analyses for *BnGLR* genes in oilseed rape. (**A**) Efficient silencing of *BnGLRs* in leaves as manifested by the dramatically reduced level of *BnGLR* transcript, which was detected by qRT-PCR analysis using *B. napus* actin (*BnActin7*) as reference gene; (**B**) representative disease symptoms on *BnGLR*-silent (*PCVA-BnGLRs*) and *BnGLR*-non silent (EV) leaves of oilseed rape cv. ZS11; (**C**) lesion areas of *BnGLR*-silenced leaves at 24 h post-inoculation with *S. sclerotiorum*; (**D**) relative fungal biomass. Data were statistically analyzed by Student’s *t*-test (n = 10) and shown as the mean ± SE. The asterisks indicate significant differences (* *p* ≤ 0.05, ** *p* ≤ 0.01, *** *p* ≤ 0.001, **** *p* ≤ 0.0001) between leaves infiltrated with PCVA-EV and *PCVA-BnGLRs*. Scale bar: 1 cm. This experiment was performed with three biological replicates, each yielding similar results.

The three *BnGLR* genes (*BnGLR12*, *BnGLR35*, and *BnGLR53*) that were expressed most highly in response to *Ss* were selected for further functional analysis in oilseed rape by reverse genetics technique Cabbage leaf curl virus (CaLCuV)-based virus-induced gene silencing (VIGS). The plants treated with empty vectors (EV) showed normal growth, suggesting that the CaLCuV-based vector infection did not obviously affect the vegetative growth of seedlings. *BnGLR* expression in the plants agro-infiltrated with *PCVA-BnGLR12*, *PCVA-BnGLR35*, and *PCVA-BnGLR53* was significantly reduced (Figure 8A), indicating that these *BnGLR* genes were efficiently silenced. Compared with the plants treated with EV, the leaves of *BnGLR35*-silenced oilseed rape plants exhibited more severe necrosis (Figure 8B) with larger lesion areas (Figure 8C) and increased relative fungal biomass of *S. sclerotiorum* (Figure 8D), indicating that the silencing of *BnGLR35* reduced oilseed rape resistance to *Ss*. In contrast, the *BnGLR12*- and *BnGLR53*-silenced plants displayed milder disease symptoms (Figure 8B) with smaller lesion areas (Figure 8C) and lower *S. sclerotiorum* biomass (Figure 8D), indicating that the silencing of *BnGLR12* and *BnGLR53* enhanced oilseed rape resistance to *Ss*. These results demonstrated that *BnGLR12*, *BnGLR35*, and *BnGLR53* play distinct roles in oilseed rape resistance to *Ss*; *BnGLR35* positively, while *BnGLR12* and *BnGLR53* negatively, regulate this resistance.

### 2.11. Arabidopsis Mutants of BnGLR Orthologs Exhibited Altered Plant Resistance against S. sclerotiorum

**Figure 9:**
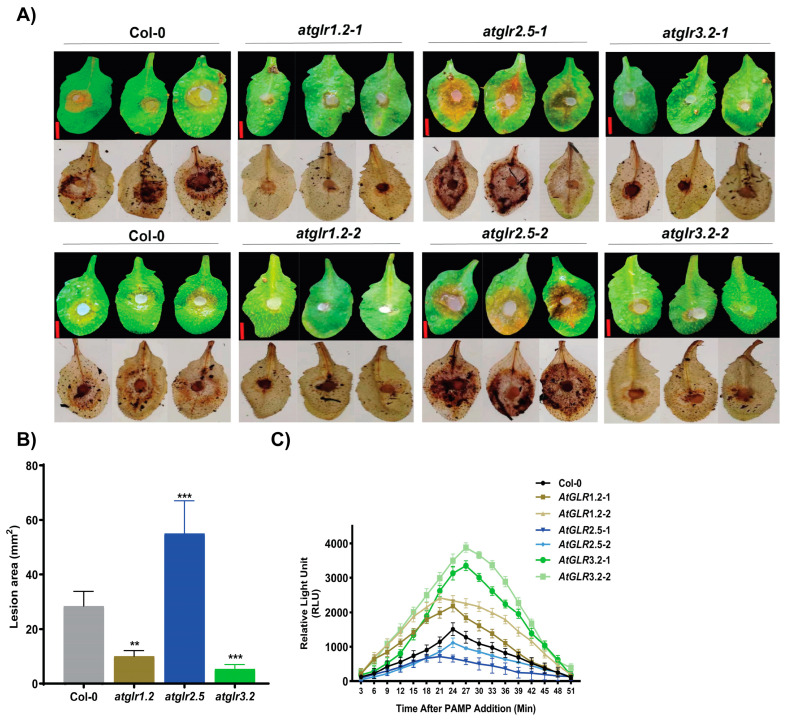
*Arabidopsis* mutants of *BnGLR* orthologs exhibited altered plant resistance against *S. sclerotiorum.* (**A**) Disease symptoms at 24 h post-inoculation (hpi). Leaves before DAB staining (upper panels) and after staining (lower panels) are shown. (**B**) Lesion areas at 24 hpi. (**C**) SsNLP1-triggered ROS in atglr plants. ROS was measured in Col-0 and *atglr* leaf disks after addition of 1 µM SsNLP1. The dynamics of ROS production within 50 min after SsNLP1 addition is shown. ROS production at each time point was calculated as mean values of total photon counts. Data were statistically analyzed by Student’s *t*-test (n = 8) and shown as the mean ± SE. The asterisks indicate significant differences (** *p* ≤ 0.01, *** *p* ≤ 0.001) of lesion areas between Col-0 and *atglr* mutants. Scale bar: 1 cm. This experiment was performed with three biological replicates, each yielding similar results.

To further explore the role of *BnGLR* genes in plant resistance against *S. sclerotiorum*, inoculation analyses were performed in leaves of 4-week-old plants of six *A. thaliana* T-DNA insertion mutants of *BnGLR* orthologs, *atglr1.2* (mutant of the ortholog of *BnGLR12*), *atglr2.5* (mutant of the ortholog of *BnGLR35*), and *atglr3.2* (mutant of the ortholog of *BnGLR53*). Prior to inoculation analysis, these *Arabidopsis* mutants were confirmed by PCR analysis and only homozygous mutant plants were selected for functional analysis. Compared with the wild type Col-0 (28 mm^2^), the leaves of both lines of *atglr2.5* plants showed more severe necrosis (53 mm^2^) with more DAB staining (Figure 9A) and larger lesion areas (Figure 9B), while the leaves of two lines each of *atglr1.2* (6 mm^2^) and *atglr3.2* (8 mm^2^) plants displayed less severe necrosis with less DAB staining (Figure 9A) and smaller lesion areas (Figure 9B). It revealed that *atglr2.5* mutants exhibited increased susceptibility to *S. sclerotiorum*, while *atglr1.2* and *atglr3.2* mutants displayed reduced susceptibility compared with the wild type Col-0. In addition, compared with the Col-0 plants, *atglr3.2* and *atglr1.2* mutants manifested the enhanced generation of SsNLP1-stimulated H_2_O_2_. Conversely, the *atglr2.5* mutants exhibited reduced SsNLP1-triggered H_2_O_2_ production (Figure 9C). These findings suggest that the *GLR* genes play a crucial role in plant resistance against *Ss*, probably via modulating ROS accumulation.

## 3. Discussion

In this study, we identified 61 BnGLRs in the genome of oilseed rape cv. ZS11. A set of evidence supports them to be potentially functional GLRs. They all contained a ligand-binding domain and an ion channel domain (Table 1; Figure S2). Their ion channel domain bore the conserved motif “SYTANLTS” (Figure S1), which was reported to be involved in ion exchange or transportation through the plasma membrane in rat, *Rosaceae*, and *Arabidopsis* [9]. Furthermore, the BnGLRs were predicted to be localized to plasma membrane and their tertiary structure fitted with membrane channel morphology, as exemplified by BnGLR12, BnGLR35, and BnGLR53 (Figure 5) as reported for known other gene family (DTX) [30]. Additionally, binding pockets of seven amino acids existed in all BnGLR proteins (Table S2) as reported typical GLRs [16]. Nevertheless, whether these BnGLRs function as calcium channels awaits further experimental confirmation.

One of our findings in this study is the group-dependent characteristics of BnGLRs. Firstly, group-wide chromosomal distribution of BnGLRs is obviously uneven. Regardless of the uncertainty of the localization of *BnGLR4* (Group I) and *BnGLR34* (Group II), Group I *BnGLRs* are absent in chromosomes A03–A05, A07–A08, A10, C03–C06, and C08, Group II *BnGLRs* are lacking in chromosomes A01–A02, A06–A10, C01, and C05–C08, whereas Group III *BnGLRs* are missing in chromosomes A01-A02, A04-A06, C02, and C06 (Table 1). Secondly, the motif composition of BnGLRs differs group-wide. For example, motif 5 is absent from Group I BnGLRs while present in those of the other two groups (Figure 2). Thirdly, the gene structure of *BnGLRs* distinguishes group-wide. *BnGLR* genes of Group III carry more introns than those of the other groups. *BnGLR* genes of Group II generally contain a long first-intron, while those of the other groups do not (Figure 3). Finally, the methylation sites discriminate between the groups of BnGLRs. The methylation sites in Glu only exist in six BnGLRs of Group III. Group III BnGLRs also contain more methylation sites in Arg and Glu than other group BnGLRs (Figure 6, Table S3). Collectively, our findings clarify the differentiation of BnGLRs of different groups, which implies that the functions and mechanisms of BnGLRs of different groups may be distinct. In this context, it is intriguing that we indeed found the distinct function of *BnGLRs* from various groups—*BnGLR12* from Group I, *BnGLR35* from Group II, and *BnGLR53* from Group III—in oilseed rape resistance to the necrotrophic fungal pathogen *S. sclerotiorum* (Figure 8). Additionally, our results on chromosomal distribution, protein motif, and gene structure of BnGLRs differ in some aspects from a previous report, which documented that Group I BnGLRs distributed in chromosomes A03, A05, A07, A08, C03–C06, and C08; Group II BnGLRs existed in A02, C06, and C07; and Group III BnGLRs presented in A01, A02, and C02. Motif 5 existed in Group I BnGLRs. Group II *BnGLRs* contained more introns (2–12 introns) than other groups *BnGLRs* [14]. These differences might be due to the variety in the oilseed rape cultivars used in the two studies [14].

Interestingly, 9 out of the total 61 *BnGLRs* carried one to several CGCG-boxes (the CAMTA-binding sites in their promoters (Figure 4)). These included *BnGLR12* from Group I, *BnGLR34* and *BnGLR35* from Group II, and *BnGLR37*, *-38*, *-39*, *-53*, *-60*, and *-61* from Group III. This demonstrates that these *BnGLRs* might be directly regulated by CAMTA3, an essential transcription factor in the calcium signaling pathways. Notably, we found previously that CAMTA3 plays a role in plant disease resistance against Ss [31]. Therefore, it is likely that CAMTA3 and these GLRs coordinate in plant resistance to Ss.

PTM is one of the most important ways to regulate protein functions. We systematically analyzed four types of PTMs, including phosphorylation, sumoylation, glycosylation, and methylation, in BnGLRs. Consequently, we found that all BnGLRs carry plenty of phosphorylation and sumoylation sites, while glycosylation and methylation sites only exist in some BnGLRs (Figure 6, Table S3). Interestingly, AtGLR3.6 and AtGLR3.7 were recently reported to be phosphorylated by calcium-dependent protein kinases at Serine-856 and Serine-860, respectively, thereby leading to salt tolerance [32,33], indicating that oilseed rape GLRs are also highly likely phosphorylated at Serine. It would be interesting to examine whether GLRs are also phosphorylated at Thr and Tyr in accordance with our prediction results. It also deserves to be confirmed whether GLRs are also sumoylated, glycosylated, and/or methylated following the clues from our study.

The function of GLRs in plant resistance to pathogens has only been studied to a limited extent mainly in the model plant *Arabidopsis*. For example, Group III *AtGLRs* were reported to play a role in plant resistance against the oomycete *H. arabidopsidis* [24], fungus *B. cinerea* [25], and bacterium *P. syringae* [26]. In this study, we explored the role of *BnGLRs* in resistance to *S. sclerotiorum*, one of the most destructive pathogens in oilseed rape, which is one of the most important oil crop species. We found that three *BnGLRs*—*BnGLR12* from Group I, *BnGLR35* from Group II, and *BnGLR53* from Group III—are highly responsive to *S. sclerotiorum* infection with an increase in expression by over 15-fold at 6 hpi (Figure 7). More importantly, VIGS analyses demonstrated that *BnGLR12*, *BnGLR35*, and *BnGLR53* play distinct roles in oilseed rape resistance to *Ss*; *BnGLR35* positively, while *BnGLR12* and *BnGLR53* negatively, regulate this resistance (Figure 8). Furthermore, inoculation analysis and ROS detection employing *Arabidopsis* mutants of the *BnGLR* gene homologs (*BnGLR12*/*AtGLR1.2*, *BnGLR35*/*AtGLR2.5*, and *BnGLR53*/*AtGLR3.2*) confirmed this conclusion and extended their roles in PAMP (SsNLP1)-triggered immunity (Figure 9). The response of Group III GLR, *AtGLR3.2*, and its ortholog in *B. napus* (*BnGLR53*) are distinguished from previous studies of *AtGLRs* from Group III, in which radish *GLR* homologs of *AtGLR3.3* and *AtGLR3.4* play a positive role in resistance against *B. cinerea* and *Alternaria brassicae* [25,34], possibly due to the difference in the pathogens used for inoculation analyses. Together with these reports, our findings reveal the complex member-dependent functions of the GLR family in plant resistance to pathogens. The mechanisms underlying *BnGLR*-mediated plant immunity await further dissection.

## 4. Materials and Methods

### 4.1. Identification of BnGLR Proteins

Using AtGLR proteins as queries, BLASTP searches were carried out against sequenced genomes of green plants in the Phytozome (https://phytozome-next.jgi.doe.gov, accessed on 26 August 2020) and NCBI (http://www.ncbi.nlm.nih.gov/, accessed on 26 August 2020) databases. Using the ClustalW2 program (http://www.ebi.ac.uk/Tools/msa/clustalw2/, accessed on 15 September 2020) with default settings, all retrieved sequences were compared with AtGLR proteins and analyzed using the GeneDoc program [8].

For the measurement of physicochemical properties such as isoelectric point (pI) and amino acid (aa) composition, an online tool ProtParam server was used (http://web.expasy.org/protparam/, accessed on 12 November 2020) [14].

### 4.2. Construction of BnGLR Phylogenic Tree

MegaX software was used to align the AtGLRs and BnGLRs by applying the clustal W function. For the construction of a phylogenetic tree in Mega X (v10.2.2) [35], the maximum likelihood (ML) algorithm with 1000 bootstrap replicates and partial deletion (95% site coverage as cut-off) were used for gaps and missing data. Based on their sequence similarity with AtGLRs in the phylogenetic tree, the members of the BnGLR family were named.

### 4.3. Identification of Motifs, Domains, and Gene Structure

Conserved motifs in the BnGLR proteins were identified using the web tool Multiple Expectation Maximization for Motif Elicitation (MEME) (http://meme-suite.org/tools/meme, accessed on 29 November 2020) [28]. The parameters were as follows: the maximum number of motifs was set to 12, the motif width ranged from 6 to 50 (inclusive), and the site distribution was limited to 0 or 1 occurrence (of a contributing motif site) per sequence. Logo motif analysis was also performed to demonstrate the conservativeness of each group of BnGLR proteins using the weblogo3 (v3.7) platform [36].

The conserved domains of BnGLR proteins were predicted using the SMART [37] and CDD databases [38].

By matching the cDNA to the corresponding gene sequences, the Gene Structure Display Server (GSDS) was used [39] to analyze the CDS and exon/intron structures of the *GLR* genes.

### 4.4. Prediction of Subcellular Localization of BnGLRs

The subcellular localization of the BnGLR proteins was predicted using the CELLO [14] web tool (http://cello.life.nctu.edu.tw/, accessed on 8 December 2023).

### 4.5. Promoter Profiling of BnGLRs

To identify the potential *cis*-acting elements in BnGLRs, upstream (1.5 kb) sequence of *BnGLRs* were obtained from NCBI and were analyzed using the search scan program in the PLACE database [8].

### 4.6. Prediction of Tertiary Protein Structure of BnGLRs

A protein modeling server, Phyre2, was used to predict the tertiary protein structures of BnGLR proteins (www.sbg.bio.ic.ac.uk/phyre2, accessed on 8 January 2021). To identify the transmembrane protein channels from their 3D structures, the PoreWalker webserver was used with pdb files of BnGLR proteins (http://www.ebi.ac.uk/thornton-srv/software/PoreWalker, accessed on 12 January 2021). To validate their secondary structures, the Protter online tool was used (http://wlab.ethz.ch/protter, accessed on 15 January 2021) [40].

### 4.7. Prediction of Amino Acid Ligands Interacting with BnGLR Proteins

To visualize the predicted physical interactions between amino acid ligands and receptor BnGLR proteins, PDB images of receptor proteins and amino acid ligands (Ala, Asn, Cys, Glu, Gly, Met, and Ser) were obtained from Phyre2 (Protein Homology/Analogy Recognition Engine V 2.0) and Pubchem (https://pubchem.ncbi.nlm.nih.gov/, accessed on 8 January 2021), respectively. Interactions were predicted by using the online tool Patchdock [41] and the complex (receptor-ligand complex) was obtained in PDB format, and the complex PDB image was visualized using LIGPLOT (v2.2) software for identification of binding pocket positions [42].

### 4.8. Post-Translational Modifications in BnGLR Proteins

The sites of PTMs, including methylation at lysine, arginine, and glutamate; N-glycosylation at asparagine; phosphorylation at serine, threonine, and tyrosine; and sumoylation were predicted by using online bioinformatics tools given below as described [43].

http://masa.mbc.nctu.edu.tw/, accessed on 20 January 2021 (Methylation);

http://www.cbs.dtu.dk/services/NetOGlyc/, accessed on 25 January 2021 (Glycosylation);

http://www.cbs.dtu.dk/services/NetPhos/, accessed on 29 January 2021 (Phosphorylation);

http://sumosp.biocuckoo.org/online.php, accessed on 2 February 2021 (Sumoylation).

### 4.9. Plant Materials and Pathogen Inoculation Analysis

*Brassica napus* plants were grown in growth cabinets at 23 °C under a 14 h/10 h light/dark photoperiod [36]. All *Arabidopsis thaliana* plants used in this study were of Col-0 ecotype background and were grown at 16 h photoperiod and 70% humidity at 21 ± 1 °C in the growth chamber [44]. The following mutants were used in this study and were obtained through the AraShare (Fuzhou, China): *atglr1.2* (SALK_136614C, SALK_053535C), *atglr2.5* (SALK_078407C, SALK_050593C), and *atglr3.2* (SALK_063873C, SALK_133700C). Homozygous plants were screened based on growth on selectable medium and PCR using a combination of gene-specific and transposon- or T-DNA-specific primers (Table S1).

Plant leaves were inoculated using *Sclerotinia sclerotiorum* (*Ss*) mycelial plugs. Fresh sclerotia of *Ss* strain UF1 were cultured at 23 °C on potato dextrose agar medium (PDA) to produce mycelia, which were transferred to new PDA plates and grown for 2 days. The PDA plugs containing young *Ss* mycelia were punched to inoculate the leaves of 30 d old plants [36]. Area of disease lesions was measured using the ImageJ (v1.54e) software [36]. For disease resistance evaluation, at least five plants for each genotype were examined and the experiments were conducted three times independently.

### 4.10. Gene Expression Analysis

A total of 10 *BnGLR* genes including *BnGLR1*, *-5*, *-8*, and *-12* (Group I), *BnGLR21*, *-26*, and *-35* (Group II), and *BnGLR37*, *-53*, and *-59* (Group III) were selected representing each group for expression analysis. One-month old oilseed rape leaves were sampled at 0, 3, 6, and 12 h post-inoculation with *S. sclerotiorum.* Total RNA was extracted using Trizol reagent (Vazyme, Nanjing, China) following the previous study [45]. Quantitative real-time PCR (qRT-PCR) was performed using the StepOne Real-Time PCR system (Applied Biosystems, Waltham, MA, USA) with SYBR Green PCR Master Mix (TaKaRa, Dalian, China). The relative fold changes were calculated using the 2^−∆∆Ct^ method as previously described [45], with two technical replicates for each of the three biological replicates. The housekeeping gene *BnActin7* was used as an internal control. Primers used for qRT-PCR are listed in Table S1.

### 4.11. Cabbage Leaf Curl Virus (CaLCuV)-Induced Gene Silencing Manipulation Procedure

The VIGS experiment with cabbage leaf curl virus was carried out as previously reported [46], with a few adjustments. (i) The gene-specific sequences of *BnGLR12*, *BnGLR35*, and *BnGLR53* were selected to ensure gene-specific silencing. Primers containing the restriction site (*Xba* I) and homologous arms were designed (Table S1). (ii) Using first-strand cDNA from the ZS11 cultivar, the target sequences of *BnGLRs* were amplified. The digested material was kept at 37 °C overnight to obtain the digested PCVA vector (digested with *Xba* I) (NEB, Ipswich, MA, USA). Using the ClonExpress Entry One Step Cloning Kit (Vazyme), the target sequence and the digested PCVA were ligated for 15 min at 50 °C. (iii) After transforming the ligation mix into competent DH5α cells (Invitrogen, Darmstadt, Germany), the cells were plated on LB plates containing antibiotics (kanamycin at 50 mg/mL) at 37 °C for overnight. A single colony was chosen, shaken well in liquid LB (containing 50 mg/mL kanamycin), and then sequenced using gene-specific primers. The plasmids from empty PCVA, PCVB, *PCVA-BnGLR12*, *PCVA-35*, and *PCVA*-*53* were purified using the FastPure EndoFree Plasmid Maxi Kit (Vazyme). (iv) Using the freeze–thaw transformation technique, plasmids were transformed into *Agrobacterium tumefaciens* (GV3101). Following that, single colonies were chosen and shaken for 48–72 h at 28 °C in liquid LB (containing 50 mg/mL of kanamycin and 50 mg/mL of rifampicin). The *BnGLRs* were sequenced using gene-specific primers and primers A and B for the PCVA and PCVB empty vectors, respectively, to confirm that the constructed vector was effectively transformed into *Agrobacterium*. A mixture of *Agrobacterium* culture and glycerol (1:3) was placed in a 2 mL cryotube to preserve bacteria for long-term preservation at −80 °C. For inoculation with PCVA/PCVB and *PCVA-BnGLRs*, ZS11 seedlings (2–3 leaf-stage) were used for VIGS experiments. Two-week-old oilseed rape plants were infiltrated with a 1 mL syringe and the infiltrated plants were placed in the dark for 24 h. The seedlings were then placed in a chamber for the following 15–21 days for successful silencing. To confirm whether plants were truly silent, half of the leaves were checked for *BnGLR* gene silencing by qRT-PCR. The remaining half of the same leaves were used for *Ss* inoculation analyses using mycelial plugs.

### 4.12. ROS Detection

For quantitative ROS measurement, 3 mm diameter leaf disks were immersed in 50 μL of distilled water in a 96-well plate and left in the dark overnight. A 100 μL solution comprising 100 μM luminol (Sigma-Aldrich, St. Louis, MO, USA) and 1 μg of horseradish peroxidase was used as a substitute for water. A microplate luminometer was used to measure the luminescence of H_2_O_2_ for 51 min after the addition of H_2_O_2_ elicitor, the fungal PAMP, SsNLP1 (1 μM) (TITERTEK BERTHOLD, Pforzheim, Germany), as previously described [36].

### 4.13. Statistical Analysis

All experiments were performed with three biological replicates. Data were statistically analyzed to determine the significant differences by Student’s *t*-test and shown as the mean ± SE using Graphpad Prism 8.0 [36].

## 5. Conclusions

A genome-wide analysis of *Brassica napus* cultivar ZS11 identified 61 *GLR* genes, grouped similarly to *Arabidopsis thaliana*. The BnGLR family exhibited some group-dependent characteristics such as chromosomal distribution, amino acid sequence motifs, gene intron number, and some PTM sites. Comprehensive bioinformatics analyses supported these BnGLRs as functional ion channels. BnGLRs were potential amino acid receptors carrying multiple PTM sites, including phosphorylation and sumoylation sites and probably also glycosylation and methylation sites. Functional assays demonstrated the positive role of *BnGLR35* and the negative role of *BnGLR12* and *BnGLR53* in oilseed rape resistance against the destructive necrotrophic pathogen *Sclerotinia sclerotiorum*. These findings advance our understanding of *GLR* family functions in plant immunity; nevertheless, functions of more *BnGLR* genes in plant immunity need to be clarified. Mechanisms underlying *BnGLR*-dependent plant immunity await further dissection. How plants elaborate the *BnGLR* genes with distinct roles in resistance to counter the pathogens represents one of the intriguing questions to be addressed in the near future.

## Figures and Tables

**Table 1:** BnGLRs identified in this study.

Protein	Accession	Protein Size (aa)	Localization	Exon/Intron	Mol. Weight (Da)	Chromosomal Position	Isoelectric Point (pI)	Domains	
								SMART Database	CDD Database
BnGLR1	XP_013666141.1	643	Plasma membrane	5/4	72,014	ChrC09	9.26	3TMD, 3LCR	PBP1, PBP2
BnGLR2	XP_013666200.1	644	Plasma membrane	5/4	72,135	ChrA10	9.44	3TMD, 2LCR	PBP1, PBP2
BnGLR3	XP_013720491.1	644	Plasma membrane	5/4	72,155	ChrA10	9.44	3TMD, 2LCR	PBP1, PBP2
BnGLR4	XP_013666142.1	656	Plasma membrane	5/4	73,622	ChrCnn_random	9.59	3TMD, 2LCR	PBP1, PBP2
BnGLR5	XP_013660587.1	802	Plasma membrane	5/4	89,788	ChrA01	7.69	4TMD, 2LCR	PBP1, PBP2
BnGLR6	XP_013745308.1	795	Plasma membrane	6/5	89,191	ChrC01	7.04	4TMD	PBP1, PBP2
BnGLR7	XP_013700213.1	833	Plasma membrane	6/5	93,803	ChrC01	6.34	4TMD, 3LCR	PBP1_GABAb_receptor_plant, PBP2
BnGLR8	XP_013724384.1	859	Plasma membrane	5/4	96,549	ChrC09	6.21	5TMD, 1LCR	PBP1_GABAb_receptor_plant, Glur_Plant
BnGLR9	XP_013660762.1	869	Plasma membrane	5/4	98,155	ChrA09	7.64	4TMD, 2LCR	PBPI, Glur_Plant
BnGLR10	XP_013724386.1	845	Plasma membrane	6/5	94,723	ChrC09	6.34	5TMD, 1LCR	PBP1_GABAb_receptor_plant, PBP2
BnGLR11	XP_013660763.1	846	Plasma membrane	5/4	94,693	ChrC09	6.57	4TMD, 1LCR	PBP1_GABAb_receptor_plant, PBP2
BnGLR12	XP_013663332.1	874	Plasma membrane	4/3	97,865	ChrA06	8.41	3TMD, 3LCR	PBP1_GABAb_receptor_plant, PBP2
BnGLR13	XP_013644299.1	873	Plasma membrane	4/3	97,979	ChrC07	8.68	5TMD, 1LCR	PBP1_GABAb_receptor_plant, PBP2
BnGLR14	XP_013674287.1	867	Plasma membrane	5/4	97,316	ChrC02	7.65	5TMD, 2LCR	PBP1_GABAb_receptor_plant, PBP2
BnGLR15	XP_022564985.1	867	Plasma membrane	5/4	97,481	ChrC02	6.79	6TMD, 1LCR	PBP1_GABAb_receptor_plant, PBP2
BnGLR16	XP_013721387.1	855	Plasma membrane	5/4	96,045	ChrA02	6.99	6TMD, 1LCR	PBP1_GABAb_receptor_plant, PBP2
BnGLR17	XP_013675190.1	866	Plasma membrane	5/4	97,047	ChrA02	8.06	6TMD, 2LCR	PBP1_GABAb_receptor_plant, PBP2
BnGLR18	XP_013721377.1	873	Plasma membrane	5/4	98,377	ChrC02	6.21	3TMD, 2LCR	PBP1_GABAb_receptor_plant, PBP2
BnGLR19	XP_013677704.1	873	Plasma membrane	5/4	97,852	ChrC02	6.13	3TMD, 1LCR	PBP1_GABAb_receptor_plant, PBP2
BnGLR20	XP_013716662.2	867	Plasma membrane	5/4	97,436	ChrA03	6.93	2TMD, 2LCR, 1PBPe	PBP1_GABAb_receptor_plant, Glur_Plant, PBP2
BnGLR21	XP_013739323.1	840	Plasma membrane	7/6	94,418	ChrC03	8.37	1TMD, 1B3	PBP1_GABAb_receptor_plant, Glur_Plant, B3, PBP2
BnGLR22	XP_022574381.1	912	Plasma membrane	7/6	102,694	ChrA04	7.13	1TMD, 2LCR, 1PBPe	PBP1_GABAb_receptor_plant, Glur_Plant
BnGLR23	XP_013745928.1	585	Plasma membrane	4/3	66,027	ChrA04	8.76	2TMD, 1PBPe	Glur_Plant, PBP1, LGC
BnGLR24	XP_022545808.1	959	Plasma membrane	5/4	107,726	ChrA05	8.69	2TMD, 1PBPe	PBP1_GABAb_receptor_plant, Glur_Plant
BnGLR25	XP_013687288.1	956	Plasma membrane	5/4	108,094	ChrA04	8.53	2TMD, 2LCR, 1PBPe	PBP1_GABAb_receptor_plant, Glur_Plant
BnGLR26	XP_013744174.1	957	Plasma membrane	5/4	108,035	ChrA04	7.90	2TMD, 1LCR, 1PBPe	PBP1_GABAb_receptor_plant, Glur_Plant
BnGLR27	XP_013744172.1	925	Plasma membrane	5/4	104,404	ChrA04	6.81	2TMD, 2LCR, 1PBPe	PBP1_GABAb_receptor_plant, Glur_Plant
BnGLR28	XP_013744173.2	922	Plasma membrane	5/4	102,918	ChrA04	8.15	2TMD, 1PBPe	PBP1_GABAb_receptor_plant, Glur_Plant
BnGLR30	XP_013746034.1	659	Plasma membrane	4/3	74,709	ChrC04	5.89	4TMD	PBP1, LGC, Glur_Plant
BnGLR31	XP_022557020.1	946	Plasma membrane	5/4	106,999	ChrC04	6.19	2TMD, 1PBPe	PBP1_GABAb_receptor_plant, Glur_Plant
BnGLR32	XP_013746035.1	880	Plasma membrane	6/5	99,879	ChrC04	6.24	1TMD, 1PBPe	PBP1_GABAb_receptor_plant, Glur_Plant
BnGLR33	XP_013721832.1	946	Plasma membrane	5/4	106,780	ChrC04	6.12	2TMD, 1PBPe	PBP1_GABAb_receptor_plant, Glur_Plant
BnGLR34	XP_013699507.1	997	Plasma membrane	6/5	112,457	ChrAnn_randm	8.94	2TMD, 2LCR, 1PBPe	PBP1_GABAb_receptor_plant, Glur_Plant
BnGLR35	XP_013717843.1	947	Plasma membrane	6/7	106,852	ChrC09	9.06	2TMD, 1PBPe	PBP1_GABAb_receptor_plant, Glur_Plant
BnGLR36	XP_013678599.1	914	Plasma membrane	5/4	103,158	ChrC02	6.09	3TMD, 3LCR	PBP1_GABAb_receptor_plant, Glur_Plant, LGC
BnGLR37	XP_013701564.1	921	Plasma membrane	6/5	103,095	ChrA08	8.57	1TMD, 2LCR, 1PBPe	PBP1_GABAb_receptor_plant, Glur_Plant
BnGLR38	XP_022545804.1	929	Plasma membrane	7/6	103,972	ChrA08	8.57	1TMD, 2LCR, 1PBPe	PBP1_GABAb_receptor_plant, Glur_Plant
BnGLR39	XP_013656439.1	929	Plasma membrane	8/7	103,924	ChrA08	8.57	1TMD, 2LCR, 1PBPe	PBP1_GABAb_receptor_plant, Glur_Plant
BnGLR40	XP_013666559.1	952	Plasma membrane	6/5	106,246	ChrA10	8.34	1TDM, 2LCR, 1PBPe	PBP1_GABAb_receptor_plant, PBP2, LGC
BnGLR41	XP_013696828.1	952	Plasma membrane	5/4	106,246	ChrC05	7.94	2TMD, 2LCR, 1PBPe	PBP1_GABAb_receptor_plant, PBP2, LGC
BnGLR42	XP_013664803.1	884	Plasma membrane	6/5	99,044	ChrA09	6.36	1TDM, 1LCR, 1PBPe	PBP1_GABAb_receptor_plant, PBP2, LGC
BnGLR43	XP_013737627.1	894	Plasma membrane	6/5	100,672	ChrC04	6.44	1TDM, 2LCR, 1PBPe	PBP1_GABAb_receptor_plant, PBP2
BnGLR44	XP_013737625.1	952	Plasma membrane	6/5	106,516	ChrC04	7.16	1TMD, 2LCR, 1PBPe	PBP1_GABAb_receptor_plant, PBP2
BnGLR45	XP_013748685.1	945	Plasma membrane	6/5	105,775	ChrA10	7.56	2TMD, 3LCR, 1PBPe	PBP1_GABAb_receptor_plant, Glur_Plant
BnGLR46	XP_013683371.1	944	Plasma membrane	6/5	105,706	ChrC05	6.33	1TMD, 3LCR, 1PBPe	PBP1_GABAb_receptor_plant, Glur_Plant
BnGLR47	XP_013683372.1	886	Plasma membrane	6/5	99,953	ChrC03	6.10	1TDM, 2LCR, 1PBPe	PBP1_GABAb_receptor_plant, Glur_Plant
BnGLR48	XP_013734086.1	885	Plasma membrane	6/5	99,802	ChrA07	6.48	1TDM, 2LCR, 1PBPe	PBP1_GABAb_receptor_plant, Glur_Plant
BnGLR49	XP_013734084.1	943	Plasma membrane	6/5	105,585	ChrC05	6.89	1TMD, 3LCR, 1PBPe	PBP1_GABAb_receptor_plant, Glur_Plant
BnGLR50	XP_013727017.1	912	Plasma membrane	5/4	101,668	ChrA07	7.97	1TDM, 1LCR, 1PBPe	PBP1_GABAb_receptor_plant, Glur_Plant, LGC
BnGLR51	XP_013692909.1	912	Plasma membrane	5/4	101,543	ChrC07	7.26	1TMD, 2LCR, 1PBPe	PBP1_GABAb_receptor_plant, Glur_Plant, LGC
BnGLR52	XP_013751706.1	913	Plasma membrane	7/6	101,458	ChrC01	8.54	1 TMD, 1PBPe	PBP1, GABAb_receptor_plant, Glur_Plant
BnGLR53	XP_013670580.1	912	Plasma membrane	7/6	101,572	ChrC01	8.55	1 TMD, 1PBPe	PBP1_GABAb_receptor_plant, Glur_Plant, LGC
BnGLR54	XP_013663029.1	891	Plasma membrane	7/6	99,380	ChrC08	8.77	1TMD, 1LCR, 1PBPe	PBP1_GABAb_receptor_plant, Glur_Plant
BnGLR55	XP_013663027.1	906	Plasma membrane	7/6	100,844	ChrC08	8.53	1TMD, 1LCR, 1PBPe	PBP1_GABAb_receptor_plant, Glur_Plant
BnGLR56	XP_013662917.1	891	Plasma membrane	7/6	99,338	ChrA09	8.65	1TDM, 1LCR, 1PBPe	PBP1_GABAb_receptor_plant, Glur_Plant
BnGLR57	XP_013734089.1	921	Plasma membrane	7/6	103,167	ChrA03	8.76	1TMD, 1LCR, 1PBPe	PBP1_GABAb_receptor_plant, Glur_Plant
BnGLR58	XP_013683373.1	921	Plasma membrane	7/6	102,816	ChrC03	8.60	1TMD, 1LCR, 1PBPe	PBP1_GABAb_receptor_plant, Glur_Plant
BnGLR59	XP_022557765.1	1068	Plasma membrane	8/7	120,615	ChrC09	8.90	1TMD, 1PBPe	PBP1, Glur_Plant
BnGLR60	XP_013737629.1	921	Plasma membrane	8/7	102,807	ChrC04	7.95	1TMD, 2LCR, 1PBPe	PBP1_GABAb_receptor_plant, Glur_Plant
BnGLR61	XP_013737628.1	928	Plasma membrane	8/7	103,572	ChrC04	8.18	1TMD, f2LCR, 1PBPe	PBP1_GABAb_receptor_plant, Glur_Plant

Abbreviations: BnGLR—*B. napus* glutamate receptor like proteins; Chr—chromosome; TMD—transmembrane domain; LCR—low complexity region; PBPe—bacterial periplasmic substrate binding proteins domain 1; PBP1—periplasmic binding protein type 1; PBP2—periplasmic binding protein type 2; GABAb_receptor—gamma-aminobutyric acid type B receptor; Glur_Plant—glutamate receptor in plants; LGC—ligand-gated ion channel; B3—B3 DNA binding domain.

## Data Availability

Data is contained within the article and Supplementary Materials.

## Supplementary Materials

The following supporting information can be downloaded at: https://www.mdpi.com/article/10.3390/ijms25115670/s1.
