# Supplementary material for: Glutamate Receptor-like (GLR) Family in Brassica napus: Genome-Wide Identification and Functional Analysis in Resistance to Sclerotinia sclerotiorum
Source: Int J Mol Sci. 2024 May 23;25(11):5670. doi: 10.3390/ijms25115670 (PMC11172227; doi:10.3390/ijms25115670)
Supplement: Supplementary file 1 [file ijms-25-05670-s001.zip › ijms-2996832-supplementary-1.pdf]

Supplementary Materials

Glutamate Receptor-lLike (GLR) Family in *Brassica napus*: Genome-Wide Identification and Functional Analysis in Resistance to *Sclerotinia sclerotiorum*

Rana Muhammad Amir Gulzar [1], Chun-Xiu Ren [1], Xi Fang [1], You-Ping Xu [2], Mumtaz Ali Saand [3] and Xin-Zhong Cai [1,4,*]

[1] Key Laboratory of Biology and Ecological Control of Crop Pathogens and Insects of Zhejiang Province, Institute of Biotechnology, College of Agriculture and Biotechnology, Zhejiang University, Hangzhou 310058, China
[2] Centre of Analysis and Measurement, Zhejiang University, 866 Yu Hang Tang Road, Hangzhou 310058, China
[3] Department of Botany, Shah Abdul Latif University, Khairpur 66020, Sindh, Pakistan
[4] Hainan Institute, Zhejiang University, Sanya 572025, China
* Correspondence: xzhcai@zju.edu.cn

A) Supplementary Figures

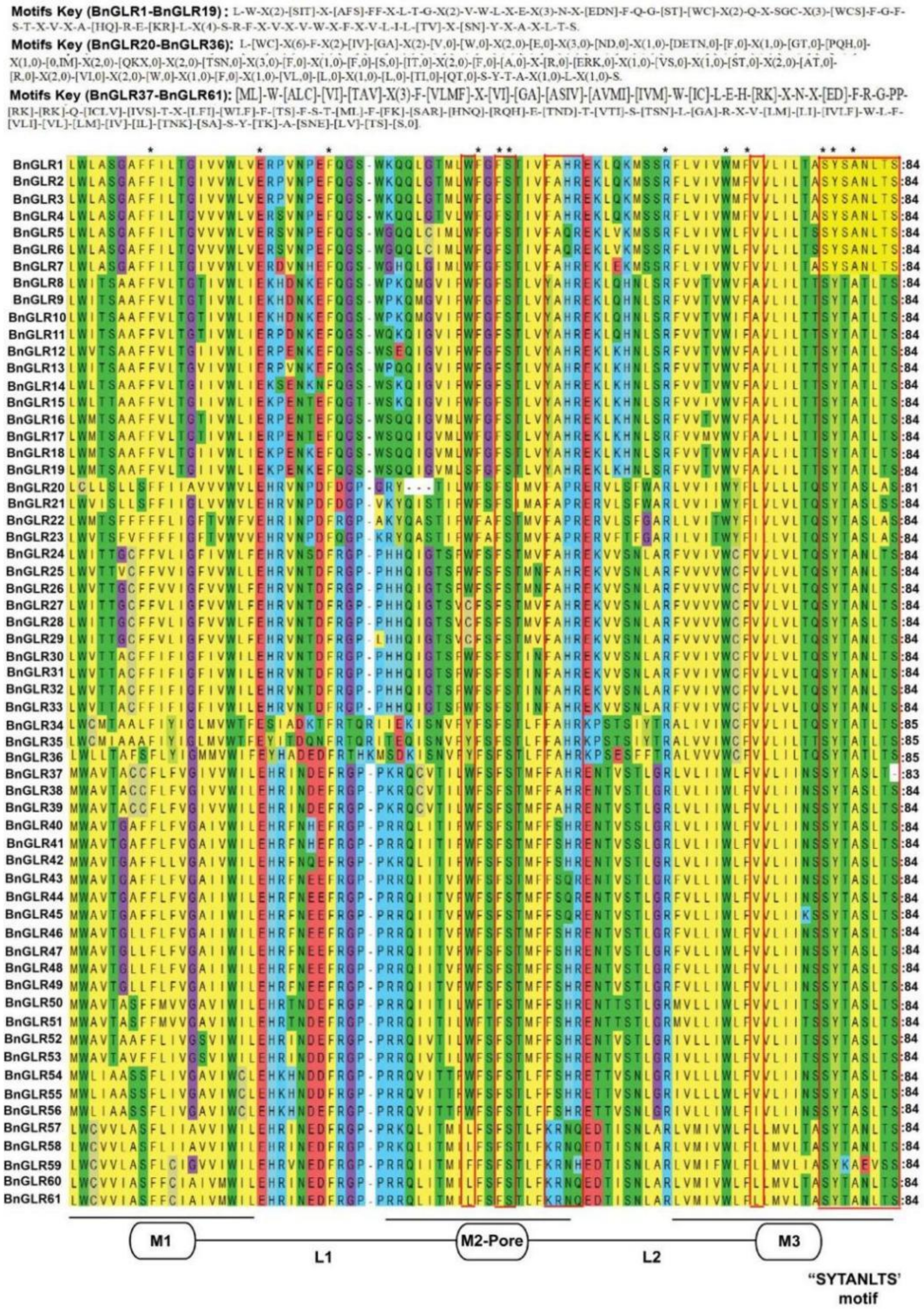

Figure S1. Alignments of the amino acid sequences of ion channel domains from 61 BnGLRs. The sequences were aligned using ClustalW. The five red outlined boxes represented channel-lining positions as determined for AMPA receptors. The residues that determine the selective transduction of cations were highly uniform among BnGLRs. Asteriks are shown on all highly conserved amino acids. In the square brackets "[]" are the amino acids allowed in this position of the motif; "X" represents any amino acid and the round brackets "()" indicate the number of amino acids. M1, first transmembrane domain; M2, Second transmembrane domain; M3, third transmembrane domain; L1 and L2 are loops region. The black asterisks above the alignment indicate 100% conservation among all *Brassica napus* GLRs. The BnGLR-specific motifs were aligned by ClustalW and generated by MEGA X program.

Gene	SMART Domains
*At*GLR1.1	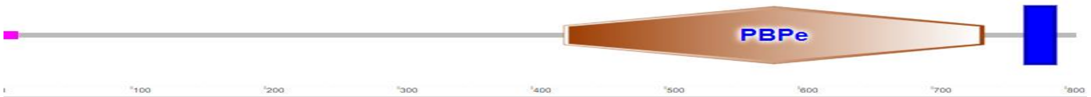
*At*GLR1.2	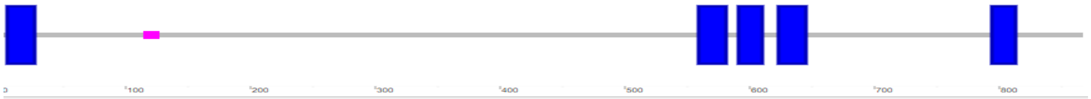
*At*GLR1.3	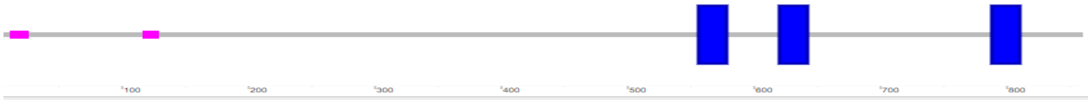
*At*GLR1.4	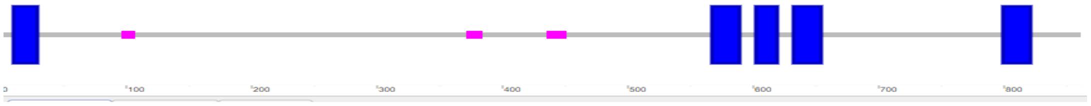
*At*GLR2.1	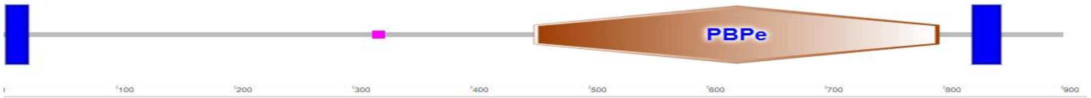
*At*GLR2.2	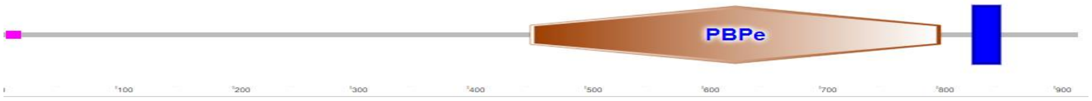
*At*GLR2.3	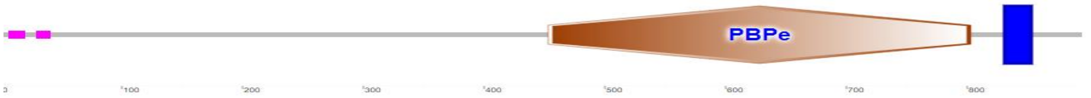
*At*GLR2.4	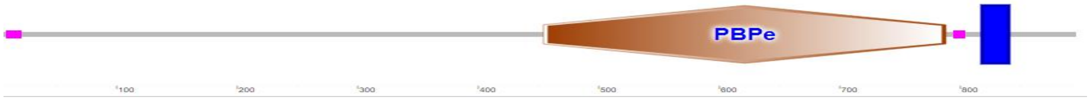
*At*GLR2.5	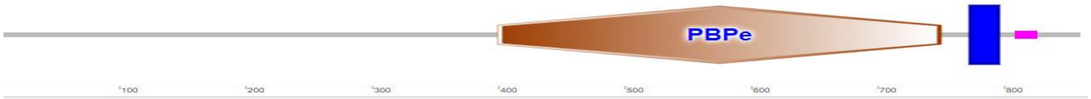
*At*GLR2.6	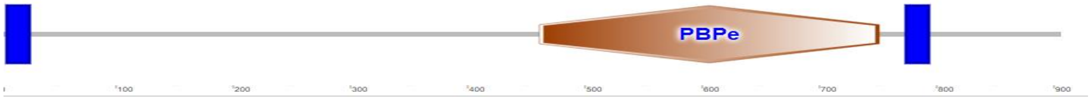
*At*GLR2.7	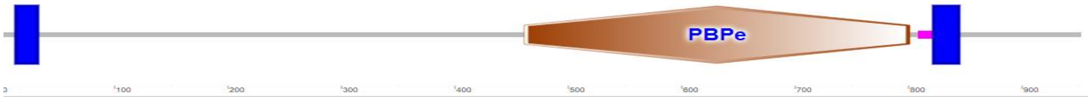
*At*GLR2.8	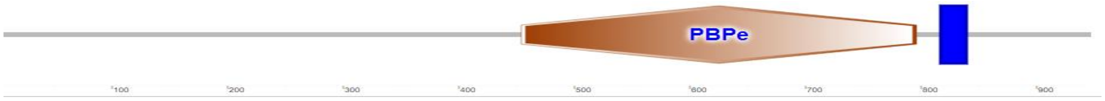
*At*GLR2.9	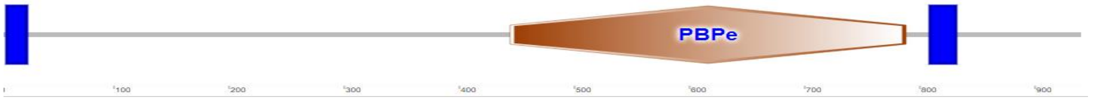
*At*GLR3.1	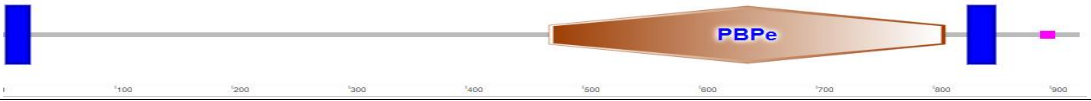

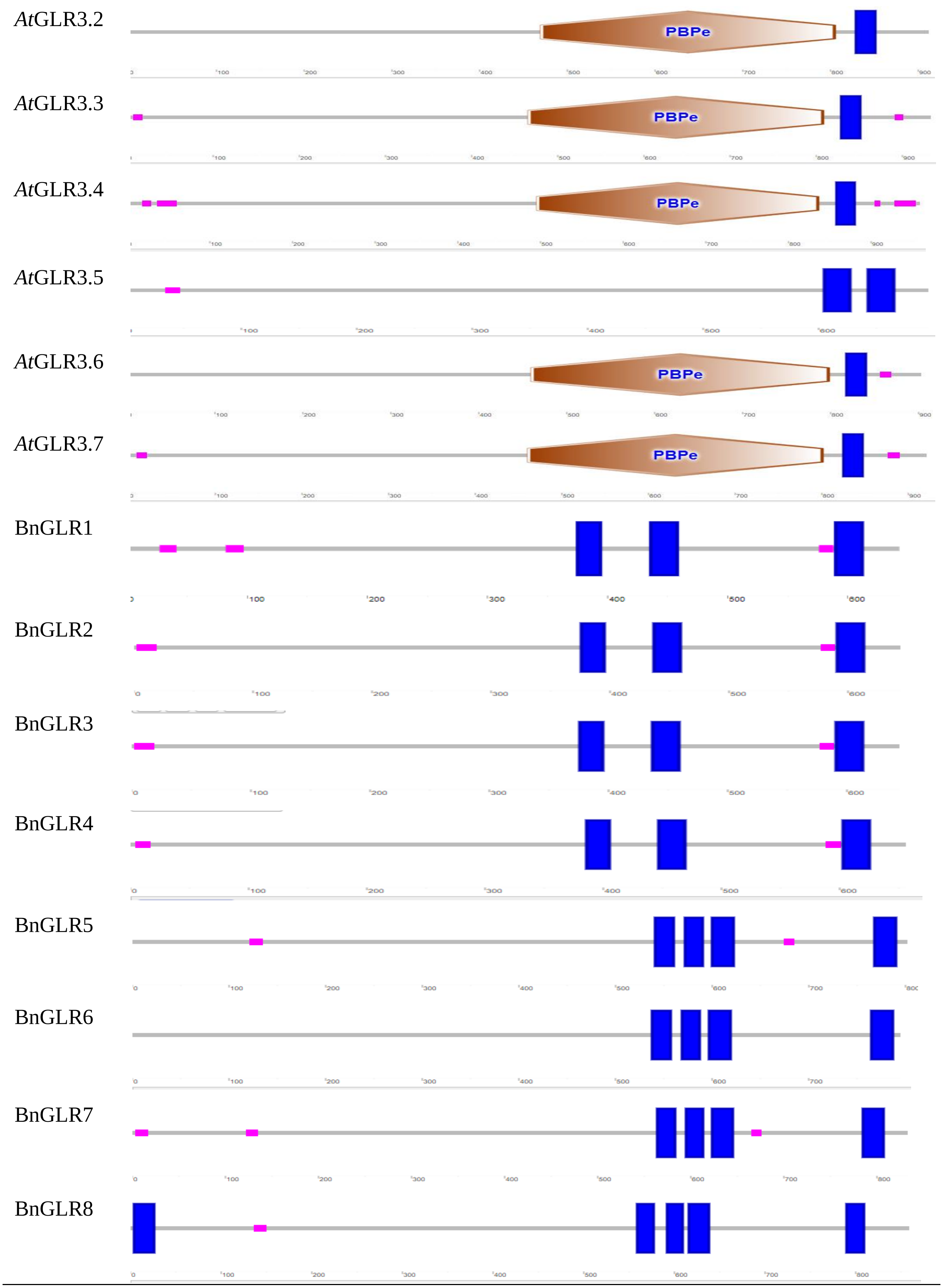
AtGLR3.2
PBPe
AtGLR3.3
PBPe
AtGLR3.4
PBPe
AtGLR3.5
AtGLR3.6
PBPe
AtGLR3.7
PBPe
BnGLR1
BnGLR2
BnGLR3
BnGLR4
BnGLR5
BnGLR6
BnGLR7
BnGLR8

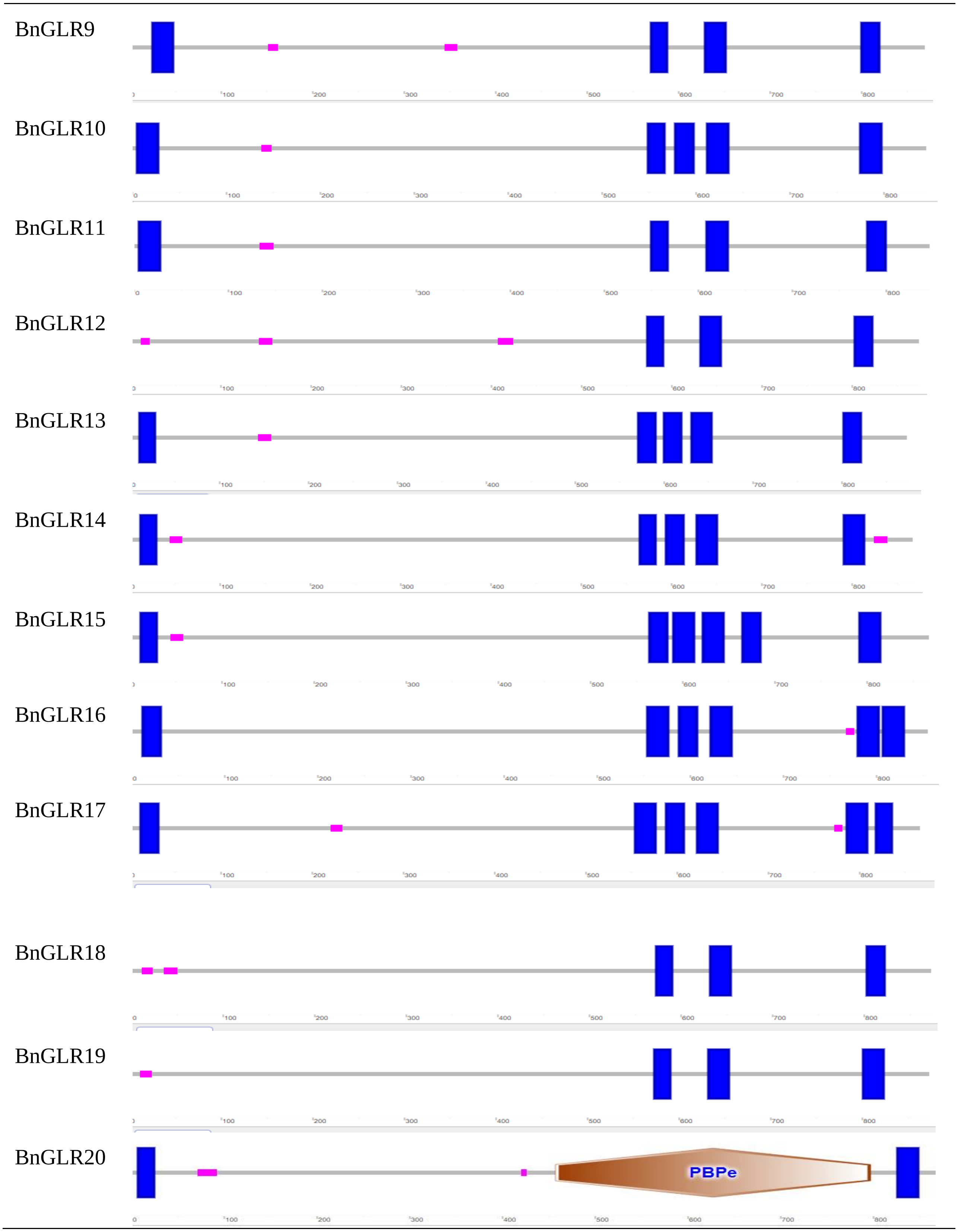
BnGLR9
BnGLR10
BnGLR11
BnGLR12
BnGLR13
BnGLR14
BnGLR15
BnGLR16
BnGLR17
BnGLR18
BnGLR19
BnGLR20
PBPe

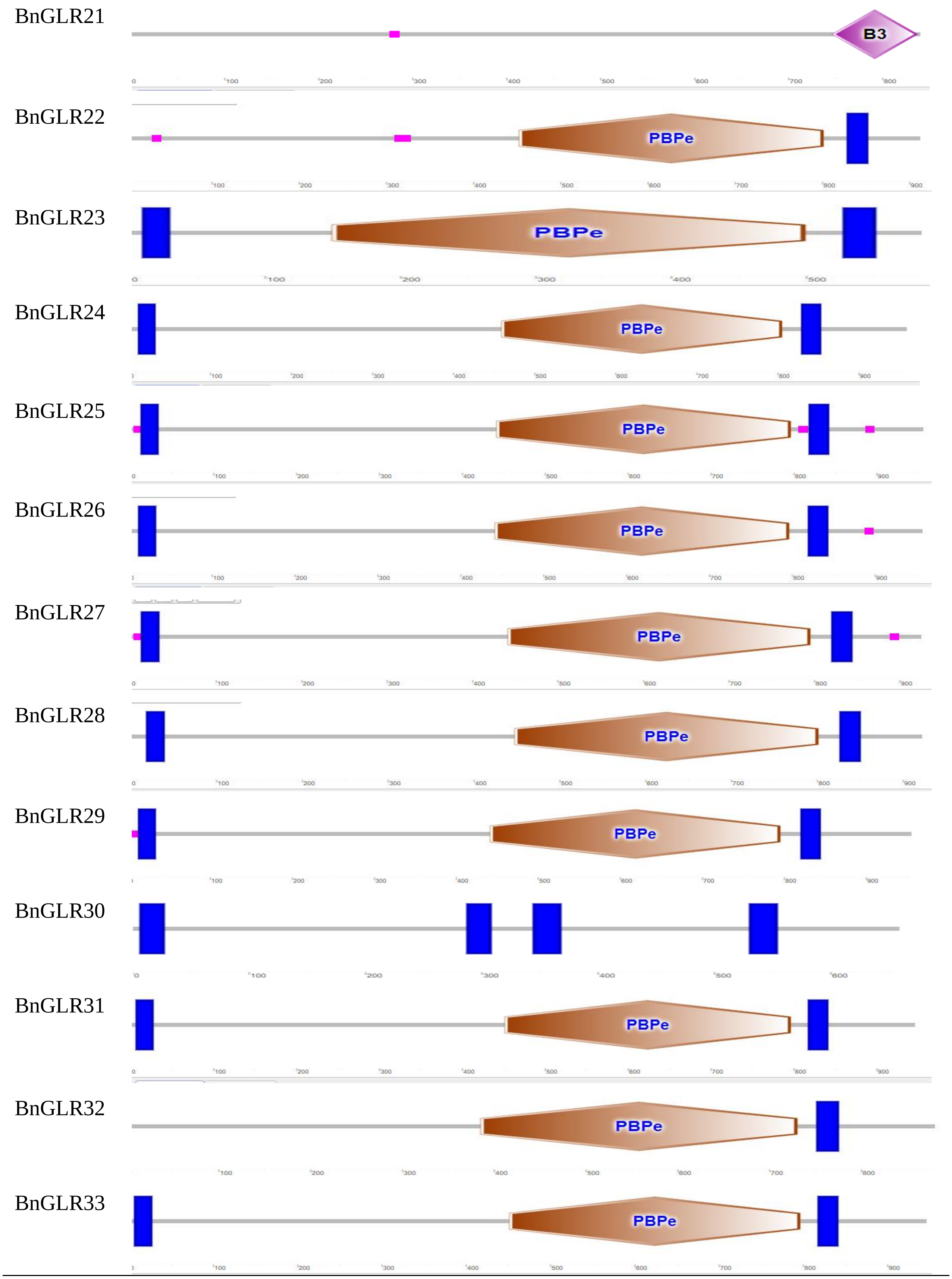
BnGLR21
B3
BnGLR22
PBPe
BnGLR23
PBPe
BnGLR24
PBPe
BnGLR25
PBPe
BnGLR26
PBPe
BnGLR27
PBPe
BnGLR28
PBPe
BnGLR29
PBPe
BnGLR30
BnGLR31
PBPe
BnGLR32
PBPe
BnGLR33
PBPe

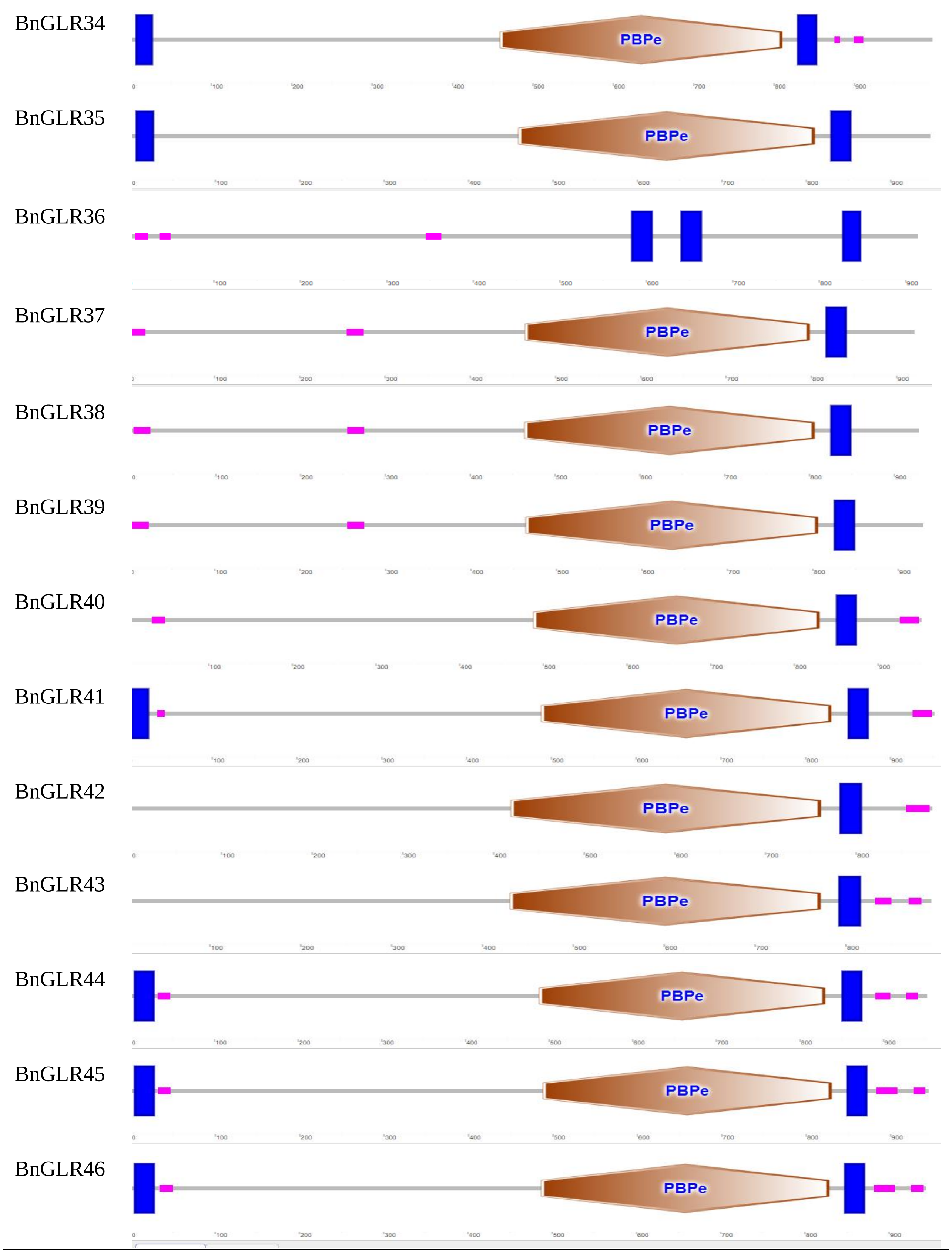
BnGLR34
PBPe
BnGLR35
PBPe
BnGLR36
BnGLR37
PBPe
BnGLR38
PBPe
BnGLR39
PBPe
BnGLR40
PBPe
BnGLR41
PBPe
BnGLR42
PBPe
BnGLR43
PBPe
BnGLR44
PBPe
BnGLR45
PBPe
BnGLR46
PBPe

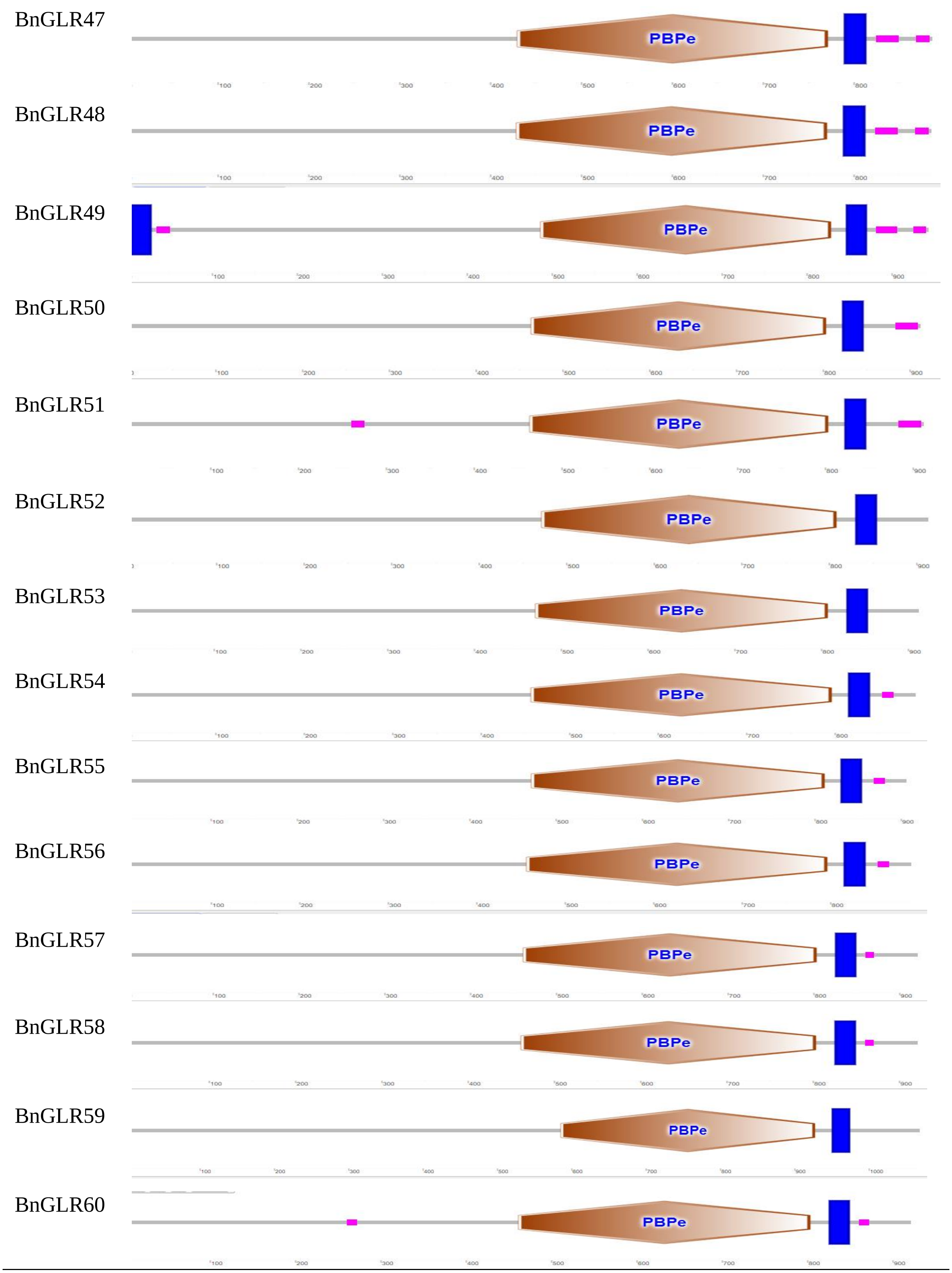
BnGLR47
PBPe
BnGLR48
PBPe
BnGLR49
PBPe
BnGLR50
PBPe
BnGLR51
PBPe
BnGLR52
PBPe
BnGLR53
PBPe
BnGLR54
PBPe
BnGLR55
PBPe
BnGLR56
PBPe
BnGLR57
PBPe
BnGLR58
PBPe
BnGLR59
PBPe
BnGLR60
PBPe

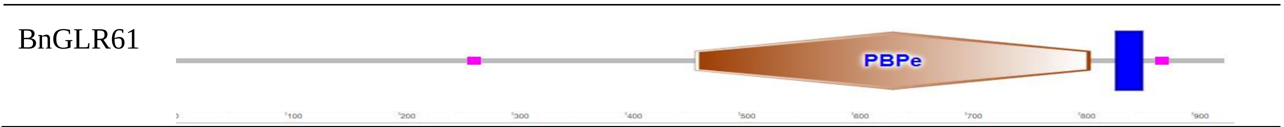

Figure S2. Domains of BnGLR proteins identified by using SMART database. All BnGLRs contained similar type of domains. The pictures were downloaded from SMART database.

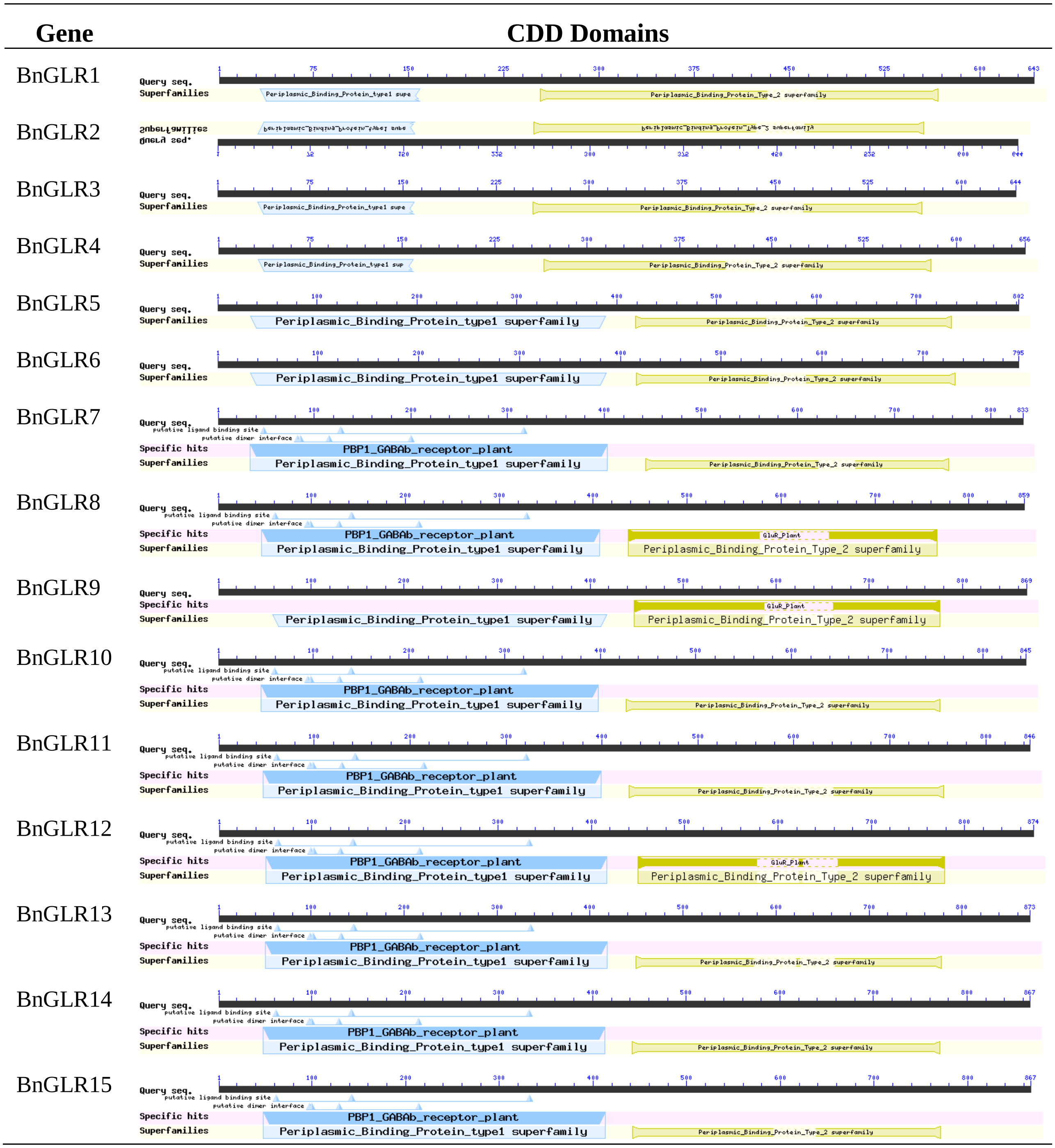

BnGLR16

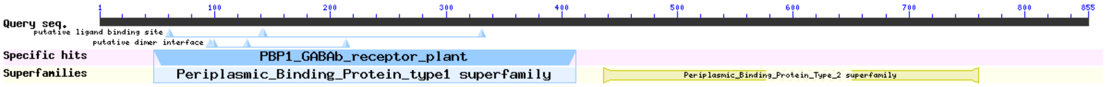

BnGLR17

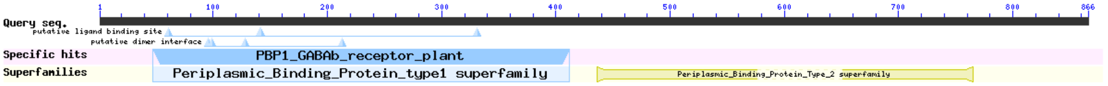

BnGLR18

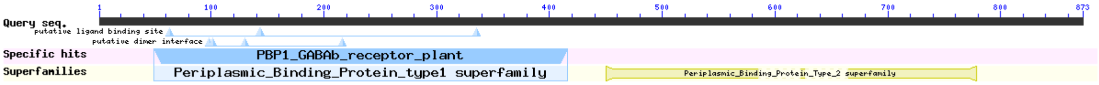

BnGLR19

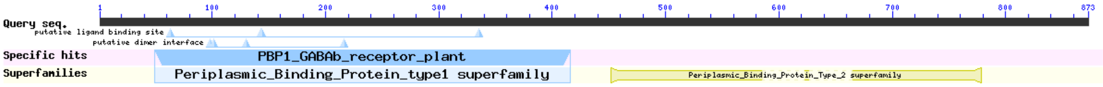

BnGLR20

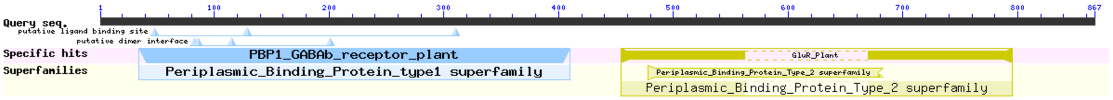

BnGLR21

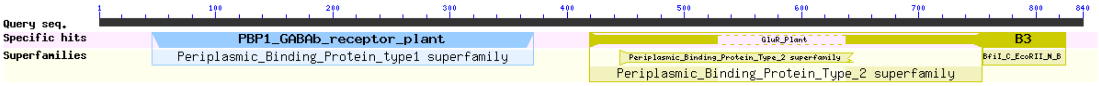

BnGLR22

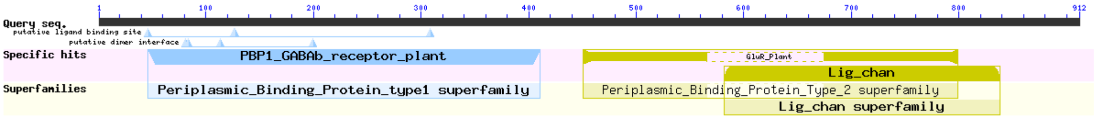

BnGLR23

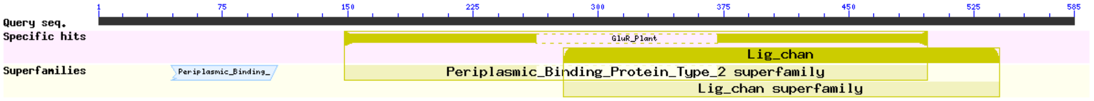

BnGLR24

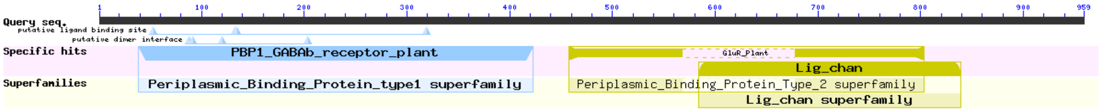

BnGLR25

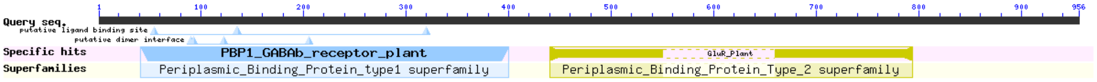

BnGLR26

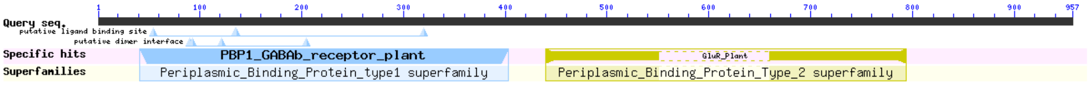

BnGLR27

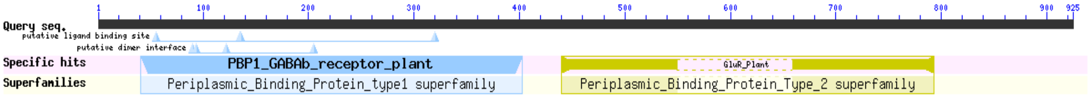

BnGLR28

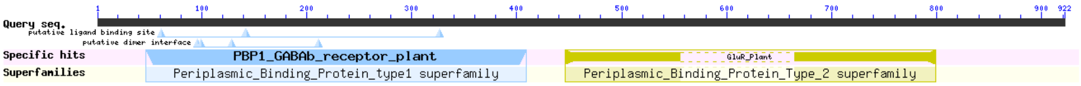

BnGLR29

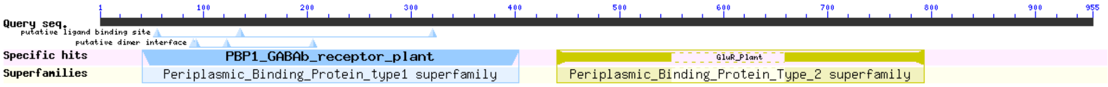

BnGLR30

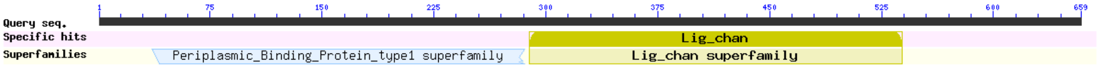

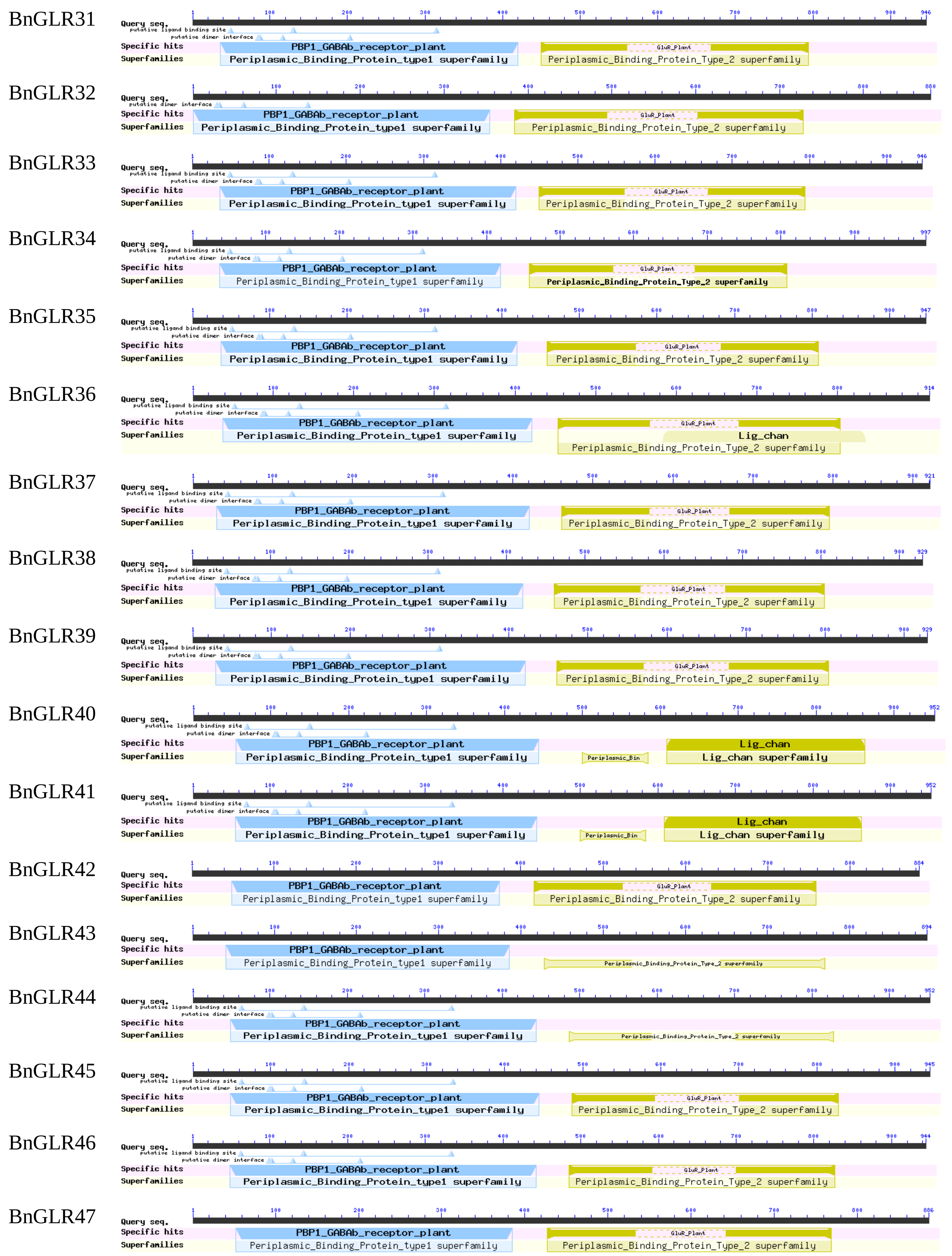
BnGLR31
BnGLR32
BnGLR33
BnGLR34
BnGLR35
BnGLR36
BnGLR37
BnGLR38
BnGLR39
BnGLR40
BnGLR41
BnGLR42
BnGLR43
BnGLR44
BnGLR45
BnGLR46
BnGLR47
Query seq.
putative ligand binding site
putative dimer interface
Specific hits
Superfamilies
PBP1_GABAb_receptor_plant
Periplasmic_Binding_Protein_type1 superfamily
GluR_Plant
Periplasmic_Binding_Protein_Type_2 superfamily
Lig_chan
Lig_chan superfamily
Periplasmic_Bin

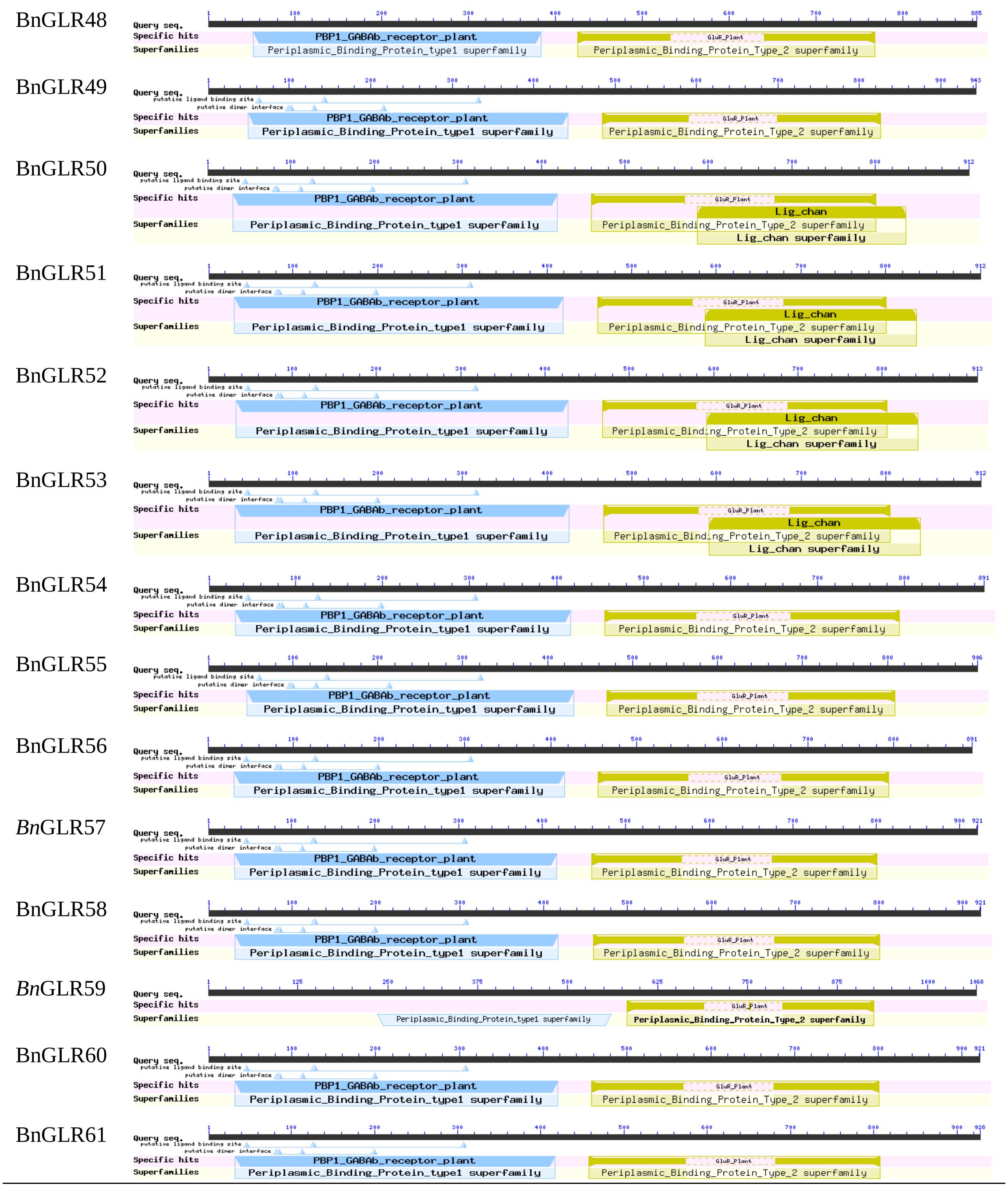

Figure S3. Domains of BnGLR proteins identified by using NCBI-CDD database. All BnGLRs contained similar type of domains. The pictures were downloaded from NCBI-CD database.

B) Supplementary Tables

Table S1. Primers used in this study.

Primer Name	Primer Sequence	Experiments
PCVA-BnGLR12-F	gctctagagcGGCTTTCAAAACCCAACTCTAG	VIGS
PCVA-BnGLR12-R	gctctagagcCCCTTCGCCTCGGAGGAAGC	
PCVA-BnGLR35-F	gctctagagcATGTCTTTTTCCGATAACATCGTGTC	
PCVA-BnGLR35-R	gctctagagcCTATGCCAAATGCATCCTCGG	
PCVA-BnGLR53-F	gctctagagcCTTCTTCAAGGCCACACGAT	
PCVA-BnGLR53-R	gctctagagcTCTCCCTTCGAGCTCGTCTC	
BnGLR12-F	GGTCTGAATATGACCGGGTTCC	VIGS-qRT Expression analysis
BnGLR12-R	AGGCTCTCCGTGAGAGTTTC	
BnGLR35-F	CCACAGCTTTCAAGCTCTAT	
BnGLR35-R	GACTTTCCCAATACGATTTG	
BnGLR53-F	CCACAGCTTTCAAGCTCTAT	
BnGLR53-R	GAGCTCGTTGCCTAGGTAAG	
PCVA-Vector Primer-F	GGGTTTCGTGGCATTTTTGTAAAT	PCVA/PCVB-Vector Confirmation primers
PCVA-Vector Primer-R	GGGGAGCAGTTATATAAGTTTCCT	
PCVB-Vector Primer-F	GCCAGCCGGAAGGGCCGAGCGCAG	
PCVB-Vector Primer-R	ATGGTTCACGTAGTGGGCCATCG	
BnGLR1-F	ATGGAGATTATGTTTTCTC	qRT-Expression analysis
BnGLR1-R	GACTCTTGTTCGATAGCCACT	
BnGLR5-F	ATGAAGAATCTGATTTCC	
BnGLR5-R	GACTCTGGTTTGATATTCA	
BnGLR8-F	ATGGAGAAGTTTGGAATTCAA	
BnGLR8-R	AAGCGCCGTTGAAGCAGAGCT	
BnGLR12-F	ATGAAGAGATTTGGCTTTCAA	
BnGLR12-R	GGAAAGAGAGCTTCTCACTAT	
BnGLR21-F	ATGACAACATCTAAAAATAC	
BnGLR21-R	GAATTGAGCCTGCAGCCAAG	
BnGLR26-F	ATGATGACAAACACCACTAC	
BnGLR26-R	AGTTTGGTAGAAATCAGACAACG	
BnGLR35-F	ATGTCTTTTTCCGATAACATC	
BnGLR35-R	GAAGCGATTATGAGTGTTAT	
BnGLR37-F	ATGAAGCTACTCTGGTCTTT	
BnGLR37-R	AACTTGAAGACGTGTCCCTACG	
BnGLR53-F	AAGCTGCAGAGGATGACGTA	

Continued

BnGLR53-R	AGAGGGGAGAGACTTGGGTC	
BnGLR59-F	CAAAACCCTAATCTGCACTC	
BnGLR59-R	AAGCAAAATGATAAGTCGGTT	
AtGLR1.2-SALK_136614C-LP	TTCGCTTCTAGCAACGAGTTC	
AtGLR1.2-SALK_136614C-RP	GATGGAAAGAGTTTGTGGTCG	
AtGLR1.2-SALK_053535C-LP	CGTTGGCTCAATGGAACTAAC	
AtGLR1.2-SALK_053535C-RP	ATCGGTTGTGTATGGAAGCTG	
AtGLR2.5-SALK_078407C-LP	GACCAAAGCTGTGTCGACTTC	
AtGLR2.5-SALK_078407C-RP	CAAGCAGATGAGGAGTTCAGG	
AtGLR2.5-SALK_050593C-LP	CCTTTTAGGTGTTCAAAGGGG	*atglrs*-LP/LB Primers
AtGLR2.5-SALK_050593C-RP	CAGCAGAAGAGAGGTACACCG	
AtGLR3.2-SALK_063873C-LP	AGATGAACAAACGTGACCACC	
AtGLR3.2-SALK_063873C-RP	TGGCTTTTTGTGGTTCTGATC	
AtGLR3.2-SALK_133700C-LP	TCCATTACTCAATTTCGGTGG	
AtGLR3.2-SALK_133700C-RP	AAACCCAAACCAAAATCATCC	
LBP1.3 (General)	ATTTTGCCGATTTCGGAAC	

Table S2. Binding of ligands with BnGLR proteins through three different bond types

Proteins	Ligands	Ligand attached to protein residues by hydrogen bond	Ligand attached to protein residues by hydrophobic bond	Ligands attached to protein by any external Bond
BnGLR1	Ala		Phe (291, 534, 536), Cys (294), Leu (302), Ala (327)	
	Asn	Leu (429), Cys (643)	Val (391), Glu (395), Phe (416), Met (432), Ser (434), Arg (435)	
	Cys	Thr (147), Ala (148), Asp (193)	Gly (153), Ile (154), Leu (157, 197), Val (191), Ser (192)	
	Gln	Ala (218)	Thr (147), Ala (148), Ile (154, 217), Leu (157, 197, 215), Ser (192)	
	Gly		Leu (256, 302), Phe (291, 536), Ala (327), Val (546)	
	Met		Thr (147), Ile (154, 217), Leu (157, 197, 215), Asn (190), Val (191), Ser (192), Ala (218)	
	Ser	Val (391), Ser (434), Cys (643)	Val (394), Glu (395), Phe (416), Leu (429), Arg (435)	
BnGLR2	Ala	Gln (322)	Asp (313), Tyr (317), Arg (323)	
	Asn	Ser (435)	Glu (396), Gln (409), Ser (420), Arg (436), Val (439)	
	Cys	Tyr (317), Gln (322)	Asp (313), Leu (318), Arg (323), Lys (325)	
	Gln	Gln (516)	Asn (265), Arg (266), Leu (270), Ile (512), Trp (567), Phe (568), Asp (572)	
	Gly	Tyr (317)	Asp (313), Gln (322), Arg (323), Lys (325)	
	Met	Gln (322)	Asp (313), Tyr (317), Arg (323)	
	Ser	Asp (313), Gln (322)	Tyr (317), Leu (318), Arg (323), Lys (325)	
BnGLR3	Ala		Leu (257, 303), Phe (292, 535), Cys (295), Ala (330), Val (547)	
	Asn	Gln (538)	Ile (161, 164), Cys (163), Leu (247), Arg (258), Asp (327)	
	Cys		Leu (257), Phe (292, 537), Cys (295), Ala (328), Val (547)	
	Gln	Asp (324)	Ile (161), Cys (163), Leu (165, 247), Arg (258), Tyr (326), Asp (327)	
	Gly		Arg (266), Val (267), Cys (288), Asp (332), Thr (350), Pro (508), Phe (533)	
	Met		Ile (161), Cys (163), Leu (165, 247), Asp (327), Gln (538)	
	Ser		Leu (257, 299, 303), Phe (292), Cys (295)	
BnGLR4	Ala		Phe (302, 548), Ala (338), Val (558)	
	Asn	Cys (305)	Leu (267, 309), Phe (302, 546, 548), Ala (340), Val (558)	

Continued

	Cys	Gln (104)	Val (37), Ala (84), Gly (103), Ser (105), Leu (112)	
	Gln	Phe (10), Ala (22), Arg (96)	Phe (14), Ser (24), Asn (25), Glu (31), Lys (33)	
	Gly	Cys (298)	Phe (359, 544, 546), Gly (545)	
	Met	Lys (33)	Phe (10, 14), Ser (24), Asn (25), Asp (26), Glu (31), Arg (96)	
	Ser		Phe (302, 548), Cys (305), Leu (309), Ala (338), Val (558)	
BnGLR5	Ala	Ser (149)	Glu (150), Asp (175), Met (229), Arg (258), Tyr (307)	
	Asn	Ser (149), Thr (256), Arg (258)	Ala (128, 257), Met (229), Ser (230), Tyr (307)	Asp (175)
	Cys	Ser (149), Tyr (307)	Glu (150), Asp (175), Met (229), Thr (256)	
	Gln	Asp (175), Tyr (307)	Ala (128, 257), His (228), Met (229), Arg (258)	Ser (149)
	Gly	Glu (731)	Lys (430), Pro (432), Phe (451, 700, 735), Asp (497), Thr (515),	
	Met	Ser (149), Glu (150), Arg (258), Tyr (307)	Ala (128, 257), Asp (175), Met (229), Ser (230), Thr (256)	
	Ser	Tyr (307)	Glu (150), Asp (175), Met (229), Ser (230), Ala (257)	
BnGLR6	Ala		Asp (172, 175), Met (230), Ser (231), Arg (259), Tyr (308)	
	Asn	Tyr (308)	Glu (150), Asp (172, 174, 175), Met (230), Ser (231), Ala (258), Arg (259)	
	Cys	Met (230)	Val (128), Asp (172, 174, 175), Ser (231), Ala (258), Arg (259), Tyr (308)	
	Gln		Gln (142), Thr (143), His (145), Trp (311), Ile (318, 356), Gly (354), Asp (355)	
	Gly		Ser (582), Gln (588), Val (601), Trp (604)	
	Met	Ser (582)	Phe (586), Val (601), Trp (604), Leu (605)	Gln (588)
	Ser	Tyr (308)	Ser (149, 231), Glu (150), Asp (172, 175), Met (230), Ala (258), Arg (259)	
BnGLR7	Ala	Thr (539)	Arg (454), Phe (475), Asp (521), Pro (696)	
	Asn		Ile (3, 286, 421), Leu (4, 288, 397), Pro (383), Arg (394), Glu (418)	
	Cys		Ile (3, 286), Leu (4, 397), His (361), Arg (394)	
	Gln		Ile (3, 286), Leu (4, 397), His (361), Pro (383), Arg (394), Glu (418)	
	Gly		Val (38), Asp (40), Ala (104), Glu (109)	
	Met		Ile (3, 286, 421), Pro (383), Arg (394), Leu (397)	Glu (418)
	Ser		Arg (454), Phe (475), Asp (521), Thr (539), Glu (752), Leu (756)	

Continued

BnGLR8	Ala	Lys (583), Pro (590), Gln (613)	Ile (577), Trp (589), Val (595), Phe (599), Arg (618)
	Asn	Ser (505)	Lys (179), Gly (428), Arg (429), Tyr (504), Lys (737)
	Cys	Arg (325)	Ser (276, 327), His (278), Trp (320), Lys (323)
	Gln	Ser (276)	Ser (275, 327), Phe (277), His (278), Trp (320), Lys (323), Arg (325), Leu (326),
	Gly	Lys (583)	Ile (577), Pro (590), Val (595), Gln (613)
	Met	Ser (505)	Lys (179, 737), Ser (180), Gly (427, 428), Arg (429), Tyr (504)
	Ser	Ser (276, 327)	Trp (320), Lys (323), Arg (325), Leu (326)
BnGLR9	Ala	Asn (869)	Leu (614, 639), Val (615), Arg (868)
	Asn	Thr (853)	Thr (650), Asn (852, 861), Asp (858), Glu (860)
	Cys	Thr (613)	Val (615, 635, 638), Tyr (616), Ala (617), Leu (639), Asn (869)
	Gln	Gly (859)	Thr (650, 853), Pro (851), Asn (852, 861), Asp (858)
	Gly	Thr (613), Leu (614), Val (615), Asn (869)	Ala (617), Val (635), Leu (639)
	Met		Phe (279), Ala (280), Gly (299), Val (300, 301), Thr (435), Arg (439), Ser (440)
	Ser	Ser (613)	Leu (614, 639), Val (615), Tyr (616), Ala (617), Arg (868), Asn (869)
BnGLR10	Ala		Gln (414, 725), Leu (421), Gly (422), Asp (500, 728)
	Asn	Gln (725), Asp (728)	Gln (414), Gly (422), Lys (425), Leu (429)
	Cys	Gln (414)	Gly (422), Leu (429, 730), Asp (500, 728), Gln (725)
	Gln	Leu (601)	Thr (559), Phe (588), Ser (591), Lys (600), Val (610)
	Gly	Thr (122), Ser (144)	Lys (123), Ile (143), Asp (187), Arg (190)
	Met	Ser (591)	Thr (559), Val (563, 710), Phe (588), Leu (601), Gln (602), Ser (606)
	Ser	Leu (421)	Gln (414), Gly (422), Leu (429), Asp (500, 728), Gln (725)
BnGLR11	Ala		Phe (73), Arg (83), Ala (329, 333)
	Asn	Asn (572)	Val (564, 585), Glu (568), Phe (589), Lys (601), Leu (602), Gln (603)
	Cys	Ser (164)	Ser (143, 163), Pro (144), Glu (165), Asp (190, 193)
	Gln	Thr (78)	Leu (70), Phe (73), Arg (83), Ala (329, 333, 336),
	Gly		Glu (112), Pro (137), Leu (332, 345), Thr (344), Ile (349)
	Met	Asp (190)	Pro (144), Asp (161, 193), Ser (163, 164, 194), Glu (165)

Continued

	Ser	Phe (73)	Leu (70, 332), Arg (83), Ala (333)
BnGLR12	Ala		Val (141), Ile (158, 340, 380), Ala (343), Phe (371)
	Asn	Glu (188), Gly (216), His (252)	Thr (218), Ser (219), Met (226)
	Cys	Gln (159)	Ala (160, 343), Phe (371, 378), Ile (380)
	Gln	Arg (229)	Glu (188), Thr (218), Ser (219, 221), Leu (225), Met (226), Val (249)
	Gly		Ala (343), Phe (371, 378), Ile (380)
	Met	His (252)	Val (17, 249), Glu (188), Gly (216), Thr (218), Ser (219), Met (226)
	Ser		Val (141), Ala (160, 343), Asp (339), Ile (340, 380), Phe (371, 378, 385)
BnGLR13	Ala		Lys (215, 257), Ile (227), Arg (230), Leu (231, 254), Trp (270)
	Asn		Val (187), Lys (215), Arg (230), Leu (231, 254), Ala (258), Trp (270)
	Cys	Lys (215)	Val (187), Arg (230), Leu (231, 254), Trp (270)
	Gln		Ile (573), Ala (577), Val (580), Phe (847), Pro (853), Ser (863). Met (867)
	Gly	Ser (361)	Glu (41, 115), Val (114, 139, 357)
	Met		Trp (587), Ile (608), Gln (866)
	Ser	Lys (215)	Ile (227), Arg (230), Leu (231, 254), Trp (270)
BnGLR14	Ala		Pro (210), Ser (211, 510), Leu (232), Asp (513)
	Asn	Ser (180)	His (208), Arg (439), Gln (741), Lys (742), Gly (743), Asp (744)
	Cys	Lys (212, 228)	Ser (211, 510), Asp (513)
	Gln	Arg (351), Gln (365)	Asn (24), Val (27, 349), Ser (29), Thr (358), Ala (362)
	Gly	Ser (211)	Lys (228), Leu (232), Ser (510), Asp (513)
	Met		Leu (232), Arg (439), Gln (741), Gly (743), Asp (744)
	Ser	Asp (119)	Val (58, 63), Gly (333)
BnGLR15	Ala		Val (47, 85), Phe (74), Ala (76, 344), Thr (83), Glu (348)
	Asn	Phe (74), Val (85), Glu (348)	Tyr (75, 81), Ala (76, 344), Lys (82), Thr (83), Arg (84),
	Cys	Thr (83), Val (85)	Val (47), Phe (74), Ala (76, 344), Tyr (81), Arg (84), Ser (347)
	Gln	Ala (76), Val (85)	Phe (74), Tyr (81), Thr (83), Ala (344), Ser (347), Glu (348)
	Gly		Val (49, 67), Leu (87, 343), Ile (116), Ala (340)
	Met		Leu (71), Phe (74), Thr (83), Val (85), Ala (344)

Continued

	Ser	Ala (76), Thr (83)	Tyr (81), Ala (344), Ser (347), Glu (348)	
BnGLR16	Ala		Gln (423), Lys (436), Asp (507, 510),	
	Asn	Lys (178, 731), Ser (179)	Val (237), Gln (426)	
	Cys	Gln (423, 730)	Glu (262), Gly (419), Thr (421), Pro (425), Lys (436), Asp (507, 510), Tyr (509)	
	Gln	Thr (236)	Asp (176), Trp (177), Lys (178), Val (237), Pro (425), Gln (426), Thr (504)	
	Gly	Gly (419)	Pro (418), Gln (423)	
	Met	Lys (731)	Trp (177), Lys (178), Val (237), Pro (425), Gln (426)	
	Ser	Gly (419), Gln (423)	Glu (262), Pro (418), Asp (507), Gln (730)	
BnGLR17	Ala		Ile (572, 576), Leu (575), Thr (843)	
	Asn	Thr (533)	Arg (447), Phe (468, 765), Asp (515), Val (709)	
	Cys		Arg (447), Phe (468, 765), Asp (515), Thr (533), Ala (761)	
	Gln		Phe (565, 840), Leu (568), Thr (569), Ile (572), Val (619, 849), Ala (841), Pro (848)	Pro (846)
	Gly	Pro (846)	Phe (565), Leu (568), Thr (569), Val (619, 849), Ser (847)	
	Met		Leu (568), Phe (565, 840), Thr (569), Ile (572), Val (619, 849), Pro (846, 848)	
	Ser		Phe (565), Leu (568), Thr (569), Ile (572), Pro (846, 848), Ser (847)	
BnGLR18	Ala		Ala (77, 347), Ser (80), Tyr (82), Glu (351)	
	Asn	His (440), Gly (441), Lys (750)	Asp (178), Lys (180, 440)	
	Cys	Val (38), Glu (351)	Asn (36), Glu (37), Phe (75), Ala (77), Trp (344)	
	Gln	Ser (80), Glu (351)	Ala (77, 347), Tyr (82), Trp (344)	
	Gly	Ser (80), Tyr (82), Ala (340)	Ala (77), Asn (79), Glu (351)	
	Met	Glu (60)	Val (53, 64), Asn (55), Gly (118, 119), Leu (142), Phe (335)	
	Ser	Glu (166)	Ser (165), Ala (273), Lys (274), Phe (335)	
BnGLR19	Ala	Glu (166)	Ser (165), Ala (273), Lys (274), Phe (335)	
	Asn	Ser (144)	Glu (190), Asp (192), Phe (245, 335), Ser (246), Ala (273), Lys (274),	
	Cys	Ser (165), Glu (190)	Glu (166), Asp (192), Phe (245, 335), Ala (273), Lys (274)	
	Gln	Met (609), Thr (616), Gln (870)	Phe (580), Val (581, 866), Gly (584, 613), Leu (610)	

Continued

	Gly		Glu (166, 190), Asp (192), Phe (245), Ala (273), Lys (274)
	Met		Leu (18), Phe (75), Ala (77), Thr (82), Trp (344), Ala (347), Glu (351)
	Ser	Ser (165), Glu (190)	Glu (166), Asp (192), Phe (245, 335), Ala (273), Lys (274)
BnGLR20	Ala	Thr (409), Tyr (456)	Tyr (315), Thr (316), Pro (411), Arg (420), Asn (422)
	Asn	Gln (175), Tyr (315), Glu (453), Tyr (456)	Thr (316), Ile (317), Asn (422), Ala (424)
	Cys	Gln (175), Tyr (315)	Thr (316), Ile (317), Pro (411), Asn (422), Gln (453), Tyr (456)
	Gln	Gly (156), Thr (158)	Ile (73), Gln (133), Ala (157), His (257), Val (258), Asn (259), Asp (286), Trp (287)
	Gly	Asn (422)	Ile (317), Ala (424), Glu (453)
	Met	Thr (285)	Gln (133), Gly (156), Ala (157), Thr (158), His (257), Pro (260), Asp (286), Trp (287)
	Ser		Tyr (315), Thr (409), Gly (410), Pro (411), Arg (420), Asn (422), Ala (424), Glu (453), Tyr (456)
BnGLR21	Ala	Thr (516)	Gly (430), Phe (451), Asp (498), Arg (759)
	Asn	Thr (516)	Gly (430), Phe (451), Asp (498), Pro (703), Leu (707), Arg (759)
	Cys	Gly (430), Thr (516)	Phe (451), Pro (703), Leu (707)
	Gln	Lys (432)	Gly (430), Phe (451), Asp (498), Thr (516), Pro (703), Leu (707, 728), Arg (759)
	Gly	Lys (772)	Val (763), Asn (764, 767), Ile (766), Lys (768), Trp (783)
	Met	Thr (3)	Ser (4), Phe (86), Ala (189), Lys (190), Tyr (268)
	Ser	Arg (759)	Gly (430), Phe (451), Asp (498), Pro (703), Leu (728)
BnGLR22	Ala	Asn (32)	Asn (25, 335), Arg (27), Val (31), Phe (333)
	Asn	Asn (25, 32, 335)	Arg (27), Glu (30), Val (31, 97), Phe (333)
	Cys	Asn (25, 335)	Arg (27), Glu (30), Asn (32), Phe (333), Ser (334)
	Gln	Gln (95), Ser (118), Gly (339), Ala (347)	Ile (91), Val (120, 342), Leu (348), Gly (349), Ser (351)
	Gly		Asn (25, 32, 335), Arg (27), Phe (333)
	Met	Ser (118)	Ile (91), Gln (95), Val (120), Gly (339, 349), Ala (347), Leu (348, 350), Ser (351)
	Ser	Asn (32, 335)	Asn (25, 26), Arg (27), Val (31), Phe (333), Ser (334)
BnGLR23	Ala	Ser (322)	Ile (290), Thr (323), Val (341), Trp (344)

Continued

	Asn		Asp (261), Leu (263), Asn (514), Pro (515), Val (517), Ser (519)
	Cys	Tyr (438)	Tyr (160), Gln (388), Ser (391)
	Gln	Arg (157), Tyr (160), Ser (416)	Gln (388), Ser (391), Phe (392), Glu (435), Tyr (438)
	Gly		Val (254), Ile (393), Lys (396), Leu (397, 457), Asn (458)
	Met	Arg (157)	Pro (155), Tyr (160), Gly (206), Gln (388), Ser (391), Glu (435)
	Ser		Asp (261), Leu (263), Asn (514), Pro (515), Val (517)
BnGLR24	Ala		Val (681), Leu (684), Ile (685), Phe (708, 739, 763), Lys (709)
	Asn	Asn (352)	Ser (125, 363), Asn (353), Leu (357, 360), Gly (361)
	Cys	Asn (352)	Ile (98), Ser (125, 363), Leu (357, 360), Gly (361)
	Gln	Gln (911), Glu (926), Arg (928)	Ser (840), Phe (878), Thr (909)
	Gly	Ser (125)	Asn (352), Gly (361), Val (362)
	Met	Asn (890), Ser (891)	Leu (826), Phe (829, 833), Val (893), Glu (944), Asn (946)
	Ser	Gln (32, 126), Ser (125), Asn (352)	Val (127), Ser (363), Gly (366)
BnGLR25	Ala		Val (827, 952), Leu (831), Thr (893), Gln (954)
	Asn		Pro (597), His (598), Ser (850), Asn (851), Lys (878), Val (882)
	Cys	Leu (910), Ala (946)	Val (569, 572), Ser (895), Leu (904), Asn (911), Gly (945)
	Gln	Glu (951)	Leu (834), Val (835), Asp (871), Ser (874), His (883), Arg (950)
	Gly	Gln (373)	Asp (155), Val (160), Glu (375), Pro (397)
	Met		Leu (831), Val (835, 885), Glu (869), Asp (871), His (883), Glu (951)
	Ser	Ala (946)	Val (569), Ser (895), Pro (896), Leu (904, 910), Asn (911), Gly (945)
BnGLR26	Ala	Lys (934)	Asn (912), Ser (923), Lys (937)
	Asn		Leu (14, 374), Phe (17), Lys (142), Asp (156), Ile (370), Gln (373), Glu (375), Trp (395), Pro (397)
	Cys	Asn (912)	Arg (913), Glu (914), Ala (920), Ser (923, 925), Lys (934, 937)
	Gln	Leu (374), Glu (375)	Phe (13, 17), Lys (142), Ile (154, 370), Asp (156), Trp (395), Pro (397)
	Gly	Asn (912), Lys (934)	Ser (923)
	Met	Lys (142)	Phe (13, 17), Asp (156), Ile (370), Gln (373), Leu (374), Trp (395), Pro (397)
	Ser		Asn (912), Ala (920), Ser (923), Lys (934, 937)

Continued

BnGLR27	Ala		Met (2), Ala (164, 195), Leu (196), Val (199, 201)
	Asn		Leu (841), Glu (845), Ile (858), Lys (861), Val (901)
	Cys	Ser (857), Lys (861),	Leu (841), Tyr (844), Glu (845), Ile (858)
	Gln		Leu (823, 912), Ile (826), Ala (827), Ser (888, 889), Pro (890, 896), Glu (915), His (922)
	Gly		Met (2), Ala (164), Leu (196), Val (199, 201)
	Met	Arg (616)	Ala (614), Arg (875), Ser (876, 881), Phe (879), Lys (880)
	Ser	Met (234), Ser (261)	Phe (158, 320), Gln (159), Leu (184), Asp (235), Asn (262), Met (264)
BnGLR28	Ala	Ala (216)	Pro (214), Ser (217), Ile (221), Leu (244), Arg (247), Val (248)
	Asn		Gln (39), Ser (43), Thr (42, 344), Arg (112), His (356)
	Cys	Thr (42, 344), Ser (43), Asn (345)	Ile (46)
	Gln		Phe (58, 326), Met (92), Pro (117), Arg (118), Ser (119), Glu (189), Leu (190)
	Gly	Ser (175), Asn (409)	Ile (172, 387)
	Met	Asp (241)	Thr (56), Phe (58), Met (92), Pro (117), Leu (190)
	Ser		Pro (214), Ala (216), Ser (217), Leu (244), Val (248)
BnGLR29	Ala	Arg (905)	Trp (567), Ile (568), Leu (903), Pro (904), Asn (910)
	Asn	Arg (905)	Trp (567), Ile (568), Gly (571), Leu (903), Ser (908), Asn (910)
	Cys		Ile (178, 249), Pro (208), Glu (218), Leu (219, 222), Val (242), Lys (245)
	Gln		Ile (178, 215), Pro (208), Glu (218), Leu (219, 222), Met (234), Val (242), Lys (245)
	Gly	Cys (802), Ala (806)	Met (739), Ala (808)
	Met	Cys (919)	Asn (589), Gly (601), Val (604), Glu (845), Phe (920), Ser (939), Ile (941)
	Ser	Trp (567), Leu (903)	Ile (568), Pro (904)
BnGLR30	Ala		His (427), Leu (430, 449), Ile (431, 452), Tyr (460)
	Asn	Asn (523), Arg (524)	Asp (61), Phe (62), Glu (64), His (66, 522), Ser (521), Lys (582)
	Cys		Phe (62), Gln (376), His (522), Asn (523), Lys (582)
	Gln		Val (162, 170, 229), Arg (168), Ala (172), Ser (169), Leu (187, 191), Ile (198, 200),

Continued

	Gly		Arg (78), Ala (87, 91), Ile (103), Met (115)	
	Met		Ala (159), Val (162, 170, 229), Leu (187, 191), Ile (198),	
	Ser	Thr (434)	Leu (430, 449), Ile (431, 452), Ala (442), Tyr (460)	
BnGLR31	Ala		Met (232, 262), Pro (233, 234), Gly (237), Phe (258), Asp (260), Thr (263), Ile (266)	
	Asn	Asn (599)	Leu (594, 633), Glu (595, 925), Val (630)	
	Cys	Met (262)	Met (232), Gly (237), Phe (258), Asp (260), Ile (266)	
	Gln	Thr (148)	Leu (149), Asp (151, 260), Phe (179, 315, 394), Gly (261), Met (262)	
	Gly	Met (232)	Phe (258), Asp (260), Met (262), Ile (266)	
	Met	Glu (595)	Leu (594, 633), Val (598), Asn (599), Ala (634), Glu (925), Phe (926)	
	Ser	Pro (233)	Met (232, 262), Pro (234), Gly (237), Phe (258), Asp (260), Ile (241, 266)	
BnGLR32	Ala	Ser (45)	Val (68), Asn (276), Ser (294), Gly (297)	
	Asn	Ser (45, 294)	Pro (69), Asn (276), Tyr (296)	
	Cys	Gln (67)	Val (68), Pro (69), Leu (300), Asn (276), Ser (45), Gly (297)	
	Gln	Leu (140), Glu (141), Arg (173)	Ala (142), Gln (146, 150), Ile (147), Lys (177)	
	Gly	Asn (844)	Met (809), Phe (810), Val (855), Glu (866)	
	Met	Glu (141)	Ile (117, 147), Leu (140), Ala (142), Ser (143), Gln (146, 150), Met (166)	
	Ser		Ser (45, 294), Val (68), Asn (276), Tyr (296), Gly (297)	
BnGLR33	Ala	Leu (389), Gln (412)	Leu (149), Asn (150), Asp (151), Arg (156), Glu (388, 390), Trp (410)	
	Asn	Asp (850), Glu (944)	His (845), Phe (847), Trp (856), Leu (942), Arg (943)	
	Cys	Arg (943)	His (845), Phe (847), Asp (850), Trp (856), Thr (941), Leu (942), Glu (944)	
	Gln	Thr (941), Arg (943), Glu (944)	His (845), Phe (847), Asp (850), Ile (901), Pro (902), Leu (942)	
	Gly	His (845)	Phe (847), Leu (859), Val (863), Pro (887)	
	Met	Leu (348)	Trp (18), Ile (95), Asn (349), Leu (357), Gly (358), Ser (360)	
	Ser	Thr (532), Arg (534)	Phe (689), Lys (755), Ser (757)	Glu (732)
BnGLR34	Ala	Ser (134), Arg (147)	Gly (108), Ser (110, 131), Ala (113, 132), Glu (179)	
	Asn		Ala (204), Phe (207), Tyr (208), Ser (209), Ile (214, 241), Glu (217), Arg (241)	

Continued

	Cys	Thr (704)	Val (564), Met (686), Lys (707), Leu (708), Glu (711), Phe (744), Tyr (768)
	Gln	Val (447)	Thr (444), Leu (446, 783), Pro (448, 778), Lys (449), Asp (537), Ser (781)
	Gly	Ser (131)	Leu (46, 234), Glu (179), Phe (180, 313)
	Met		Ala (204), Ile (214, 241), Glu (217), Leu (237),Arg (240)
	Ser	Ala (204)	Phe (207), Ile (214, 241), Leu (237), Arg (240)
BnGLR35	Ala		Val (580), Leu (670, 674), Met (673), Lys (877), Asp (895)
	Asn	Lys (100)	Val (101, 125), Gln (124), Pro (126), Phe (338), Thr (340), Asp (348), Ala (359), Gly (362)
	Cys		Val (580), Leu (670, 674), Met (673), Lys (877), Asp (895)
	Gln		Val (102, 125), Ser (123, 361), Gln (124), Phe (338), Thr (340), Asp (348), Ala (359), Gly (362)
	Gly		Leu (670, 674, 826), Lys (877), Asp (895)
	Met	Gln (124)	Val (125), Pro (126), Phe (338), Thr (340), Asp (348), Ala (359), Gly (362)
	Ser	Asn (118)	Met (1), Asp (5), Asn (6), Ile (117), Leu (140), Arg (141), Ser (142), Pro (143)
BnGLR36	Ala		Val (42), Ala (89, 93), Ile (117)
	Asn		Ile (215), Glu (218), Leu (238, 242), Arg (241)
	Cys	Gly (109), Ser (132)	Asn (110), Ala (114, 133), Ser (130, 135), Phe (146)
	Gln	Ser (628)	Ile (595), Phe (631, 632), His (634), Val (647), Trp (650), Cys (651)
	Gly		Val (42), Ile (105, 117)
	Met		Ala (205), His (209), Ser (210), Ile (215), Met (234), Leu (238, 242), Arg (241)
	Ser		Val (42), Ala (89, 93), Ser (92), Ile (117)
BnGLR37	Ala		Phe (237), Ser (259, 264), Asp (263), Ala (279)
	Asn		Leu (562, 696,707), Ile (674, 684), Tyr (686), Glu (704), Val (733)
	Cys		Phe (237),Ala (254), Ser (259, 264),Leu (262), Val (281, 402)
	Gln		Leu (562, 696,707), Ile (684), Tyr (686), Phe (756), Glu (704), Val (733)
	Gly		Phe (237), Ser (259), Val(281), Leu (262)

Continued

	Met		Ile (674, 684), Tyr (686), Glu (704), Leu (707), Val (733)
	Ser		Phe (237), Ser (259, 264), Leu (262), Pro (267), Ala (279), Val (402)
BnGLR38	Ala		Val (118), Phe (339, 362), Gly (365), Leu (368)
	Asn		Ile (94), Val (118), Pro (119), Phe (339, 362), Asn (341), Gly (364, 365), Leu (368)
	Cys	Ile (94), Val (95)	Val (118), Pro (119), Phe (339, 362), Ser (351), Gly (365), Leu (368)
	Gln	Ser(338), Asn (341)	Ile (94), Val (95, 337), Phe (339, 362), Gly (365), Leu (368)
	Gly		Glu (24), Lys (25), Pro (60), Val (64), Gly (65), Asp (328), Phe (331)
	Met	Gln (847), Glu (861)	Val (621), Trp (625), Glu (860), Ser (873, 874), Leu (875)
	Ser	Val (95)	Ile (94), Pro (119), Phe (339, 362), Gly (364, 365), Leu (368)
BnGLR39	Ala	Gly(197)	Ile (209), Leu (232), Ala (235),Val(236)
	Asn	-	Gly (197), Asn (205), Ile(209), Met (212), Leu (232), Ala (235), Val (236)
	Cys		Ile (832), Val (835), Phe (883), Ser (885), Glu (889), Lys (895)
	Gln		Val (835), Ala (836), (Leu 839), Phe (883), Ser (885), Glu (889, 891)
	Gly		Leu (116), Arg (117), Val (118), Phe (339, 362)
	Met	Ser (885), Glu (891)	Ile (832), Leu (839), Phe (883), Glu (889), Lys (890, 895)
	Ser		Gly (197), Pro (200), Met (212), Leu (232), Val (236)
BnGLR40	Ala		Trp (22), Pro (51), Ser (53), Asn (55), Gln (118), Val (120)
	Asn	Asn (55), Gln (118)	Trp (22), Pro (51), Ser (53), Val (54, 120), Thr (360), Phe(361)
	Cys		Ala (111, 122, 138), Met (115), Val (137, 143),Leu (145)
	Gln	Asp (84)	Val (81), Asn (82), Gln (85), Gly (90), Thr (91), Lys (92), Ala (346), Asp (350)
	Gly	Phe (361)	Trp (22), Val (120), Thr (360)
	Met		Val (81), Asp (84), Gln (85), Gly (90), Thr (91), Lys (92), Ala (346), Asp (350)
	Ser		Trp (22), Pro (51), Ser (53), Asn (55), Val (120), Phe (361)
BnGLR41	Ala		Phe (260), Pro (282), Leu (285), Val (302), Ile (423)
	Asn		Pro (46, 51), Ser (53), Asn (55), Gln (118), Val (120), Thr (360)
	Cys	Asp (286)	Phe (260), Ala (277), Pro (282), Leu (285), Ser (287), Val (302), Ile (423)

Continued

	Gln	Gly (156), Thr (158)	Ile (73), Gln (133), His (257), Val (258), Asn (259), Asp (286), Trp (287)
	Gly	-	Phe (260), Ala (277), Leu (285), Ser (287), Val (302)
	Met	Thr (662)	Val (624, 663), Leu (627), Glu (628), His (629, 948), Ser (664, 949), Pro (952)
	Ser	Pro (282)	Phe (260), Ala (277, 290), Asp (286), Ser (287), Val (302)
BnGLR42	Ala		Ile (128, 168), Val (195, 198), Ala (199), Trp (211)
	Asn	Ser (581)	Phe (578, 582), Met (585), Glu (818), Cys (823), Arg (833)
	Cys		Met (51), Val (55,56,79), Pro (80), Phe (314)
	Gln	Gly (816)	Phe(578, 582), Trp (579, 802), Glu (818), Arg (821)
	Gly	Val (195)	Ile (128), Leu (171, 172)
	Met	-	Val (55, 56, 79), Ile (290), Thr (291, 299), Asp (303), Phe (314)
	Ser	Val (195)	Ile (128), Leu (171, 172), Val (198)
BnGLR43	Ala	-	Leu (808, 869), Phe (811), Val (815), Gly (828), Asp (829), Ile (865), Met (868)
	Asn	Ser (594)	Phe (598), Gln (600), Glu (602), Leu (613), Trp (616)
	Cys	Gln (8)	Leu (9, 13, 291), Asn (10), Trp (14), Ala (288)
	Gln	Glu (602)	Ser (594), Phe (598), Gln (600), Leu (613, 617), Trp (616)
	Gly	Arg (15)	Leu (17, 291), Ala (57), Ile (59)
	Met	Ser (594)	Gln (600), Leu (613), Trp (616)
	Ser	Gln (8), Asn (10)	Leu (13), Trp (14), Arg (15), Ala (288)
BnGLR44	Ala	Ala (891)	Ser (650), Glu (893),Lys (634), Gly (621)
	Asn	Thr (653), Glu (893), Lys (934)	Ala(622, 891), Val(646),Leu(618), Ser(650), Gly (621)
	Cys	Glu (893)	Leu (618), Gly (621), Ser (650), Lys (934), Ala (891), Val (646)
	Gln	Glu (350)	Ala (77, 346), Ser (80), Val (15, 75), Leu (82)
	Gly	Phe (154)	Phe (157),Glu (410),Gln (153), Asn (412), Lys (411)
	Met		Ile(643, 644), Arg (640),Asp (887), Glu (888), Trp (875), Lys (872)
	Ser	Ser (650), Lys (934)	Leu (618), Gly (621), Glu(893),Val(646), Ala(891), Arg (890)
BnGLR45	Ala		Ser(908), Asp (911), Glu (922), Met (926), Gln (939)
	Asn	Arg(905), Ser(908)	Phe (635),Gln (642), Ala(907), Asp(911), Glu (925),Trp(625), Arg

Continued

			(636), Lys (910)
	Cys	Ser (908)	Asp (911), Leu (912), Glu (922), Met (926), Ser (941), Gly (942)
	Gln	Lys (924)	Leu (618), Ala (622), Phe (647),Gly (621), Arg (899), Val (891)
	Gly	Asp (273)	Val (245), Asn (246), Pro (247), Gly (250), Ala (271)
	Met	Tyr (334)	Phe (59,142), Pro (119), Ala (143),Glu (191), Tyr (192)
	Ser		Ser (908, 940, 941), Asp(911), Leu (912), Glu (922), Met (926), Gln(939)
BnGLR46	Ala	Glu (883)	Glu (893), Ser (897, 907), Phe (908), Ile (912)
	Asn	Asp (924), Ser (930)	Ser (891, 939), Met (925), Gln (928),Glu (942)
	Cys	Glu (942)	Pro (637), Leu (879), Val (890), Glu (887, 892), Lys (927)
	Gln	Ser (891, 939), Thr (943)	Met (925), Gln (928, 938), Ser (930), Glu (942)
	Gly		Tyr (177), Gly (431), Ser (440), Val (441)
	Met	Ser (930, 939)	Ser (891), Asp (924), Gln (928, 938),Ala(936), Glu (942)
	Ser		Pro (637), Leu (879), Glu (887, 942), Val (890), Lys (927)
BnGLR47	Ala	Arg (765)	Ser (437), Tyr (438), Met (522), Pro (709), Leu (769)
	Asn	Arg (765)	Tyr (438), Met (522), Pro (709), Leu (769)
	Cys	Arg (765)	Tyr (438), Met (522), Pro (709), Leu (769)
	Gln	Val (56), Glu (326)	Met (51), Val (55,79),His (78), Pro (80), Lys (54), Phe (324)
	Gly	Lys (18), Ile (59)	Trp (15), Leu (17), Met (21), Ala (57, 58)
	Met		Thr (554),Trp (615), Phe (558, 596), Ser (839, 841), Arg (840)
	Ser	Arg (765)	Ser (437), Tyr (438), Pro (709), Ala (713), Leu (769)
BnGLR48	Ala	Met (230), Ser (365)	Leu (233), Gln (234), Ile (363), Asn (364)
	Asn	Ser (365)	Thr (229), Met (230), Leu (233), Gln(234), Ile (363, 406), Asn (364)
	Cys	Gln (8)	Leu (9,13), Asn (10), Trp (14,15), Ala (286)
	Gln		Met (51), Val (55,56,79), His (78), Phe (301, 323), Gly (312, 326), Glu (325)
	Gly	Ser (365)	Met (230), Gln (234), Ile (406), Trp (407), Pro (408)
	Met		Met (51), Lys (54), Val (55, 79), His (78), Phe (301, 323), Gly (312)
	Ser	Ser (365, 390), Trp (407)	Asn (364), Ile (406), Pro (408)
BnGLR49	Ala		Leu (135), Gly (370), Thr (371), Asn (372), Ile (373), Ser (379),Phe

Continued

			(381)
	Asn	Ser (379)	Met (109), Leu (135, 378), Gly(370), Thr (371), Asn (372), Ile (373), Phe (381)
	Cys	Leu (378)	Cys (18), Val (19, 380), Phe (22), Glu (134)
	Gln		Thr (611),Gly (612), Phe (653, 907), Trp (672), Val (676), Ile (679), Ala (904),
	Gly		Tyr (192), His (244), Val (245), Thr (272), Asp (273), Trp (274)
	Met	Ser (8)	Cys (4), Val (5), Phe (22), Glu (134), Leu (378)
	Ser	Gly (370)	Leu (135, 378), Thr (371), Asn (372), Ile (373), Ser (379), Phe (381)
BnGLR50	Ala	Ser (59)	Ile (30), Val (57, 324), Asp (60), Pro(61), Gly (66), Ala (321),
	Asn		Val (597, 601), Ile (881), Leu (899), Met (901)
	Cys	Ser(59), Ala (321)	Ile (30), Asp (60), Gly(66), Ser (67), Val (324)
	Gln	Ser (897)	Val (597, 601), Ala (600), Ile (881), Met (901)
	Gly	Asp (400)	Asn(276), Gly (277), Val (398), Ser(428), Trp (442), Pro (443)
	Met	-	Met (91), Asp (94), Val (95), Ser (343), Phe (358), Glu (360)
	Ser	Arg (26), Thr (335)	Ile (30), Val (96, 324), Phe (327), Leu (334, 364)
BnGLR51	Ala	Val (95, 96)	Met (91), Thr (118), Phe (336, 358), Gly (361)
	Asn	Glu (606), Ser (642)	Ile (602), Leu (605, 644), Phe (627), Ile (912)
	Cys	Thr (118)	Met (91), Val (95, 119), Pro (120), Phe (336, 358), Glu (360), Gly (361), Leu (364)
	Gln	Phe (148), Thr (257), Asn (229)	Ala (126), Met (150), Asn (174), Ser (175), Gly (178),
	Gly	Thr (118)	Val (95, 119), Phe (336, 358), Gly (361)
	Met	Lys (31)	Gly (21), Ala (22), Pro (27), Val (29, 96), Ile (30), Thr (335)
	Ser	Val (95), Thr (118)	Val (96, 119), Pro (120), Phe (336, 358), Leu (364)
BnGLR52	Ala	Ser (830)	Ala (583), Leu (672, 676), Ile (675), Arg (829)
	Asn	Lys (279), Tyr (432), Trp (450)	Ala (278), Thr (282), Val (406, 407), Gly (408), Thr (449), Pro (451)
	Cys	Tyr (432)	Lys (279), Leu (281), Thr (282), Gly (408), Pro (451), Val (406, 407)
	Gln	Lys (279, 434), Tyr (432), Pro (451)	Ala (278), Leu (281), Thr (282), Val (406,407), Thr (449), Trp (450)
	Gly		Leu (151), Val (155, 181), Gly (180), Asn (231), Thr (259)

Continued

	Met		Leu (281), Thr (282, 449), Gly (283), Val (406), Tyr (432), Lys (434), Trp (450), Pro (451)
	Ser	Lys (279), Leu (281), Val (406)	Thr (282), Val (407), Gly (408), Trp (450), Pro (451)
BnGLR53	Ala	Glu (867)	Leu (881), Arg (898), Asp (905)
	Asn		Pro (620), Glu (867), Arg (877, 898), Leu (881), Asn (904), Asp (905)
	Cys	Glu (867), Arg (897)	Leu (881), Arg (898), Asn (904), Asp (905)
	Gln	Met (92)	Asp (95), Ser (96), Val (97, 120), His (119), Pro (121), Asn (350), Phe (365), Gln (367), Gly (368)
	Gly	Phe (149)	Asp (129), Asn (175)
	Met		Ala (582), Leu (576, 671, 675, 825), Ile (674), Ser (829)
	Ser	Glu (867), Asp (905)	Leu (881), Arg (898)
BnGLR54	Ala	Ile (71), Ser (106)	Val (35), Thr (37), Asn (79), Ser (83), Glu (86), Pro (101), Arg (103)
	Asn	Lys (353)	Met (91), Leu (117, 349), Ile (119), Gly (344), Val (354), Phe (355)
	Cys	Asp (128)	Gln (102), Arg (103), Pro (104), Ala (107), Thr (127), Asp (173)
	Gln	Arg (855)	Phe (584,622), Trp (577), Leu (621), Ile (854), Arg (856)
	Gly	Asp (356)	His (12), Leu (16), Ser (24), Phe (355)
	Met		Ile (672, 682), Leu (675, 694), Glu (676), Tyr (684), Val (731), Phe (754)
	Ser	Thr (37), Ile (71), Asn (79), Ser (83, 106)	Glu (86), Pro (101), Gln (102), Arg (103)
BnGLR55	Ala	Gln (117), Ala (141)	Arg (118), Pro (119, 144), Thr (140)
	Asn		Trp (592), Ala (595), Phe (599, 637), Ile (663), Asp (905)
	Cys	Met (106), Thr (110)	Asn (37), Val (38), Lys (109), Leu (357), Gly (359), Phe (370)
	Gln	Ile (906)	Trp (592), Ala (595, 596), Phe (599, 637), Ile (663), Gln (902), Glu (904), Asp (905)
	Gly	Gly (193)	Ala (141), Pro (144), Asn (160, 192), Phe (163), Met (165)
	Met	Gly (193)	Ala (141), Pro (144), Asn (160, 192), Phe (163), Met (165)
	Ser	Ile (336)	Thr (77), Asp (337), His (339), Ile (353)
BnGLR56	Ala	Tyr (228)	Pro (104), Thr (125), Phe (148), Asp (173), Tyr (174)
	Asn	Ser (859)	Phe (614, 618), Ser (617, 853, 858), Gly (852), Ile (854), Pro (860)
	Cys	Thr (125)	Gln (102), Phe (148), Asp (173), Tyr (174, 228)

Continued

	Gln	Trp (641)	Trp (577), Ala (580), Phe (584, 622), Val (645), Ile (648), His (865)
	Gly		Leu (212), Val (238), Met (244), Trp (251)
	Met		Trp (577, 641), Ala (580, 581, 862), Phe (584), His (865)
	Ser	Tyr (228)	Gln (102), Arg (103), Pro (104, 129), Thr (125), Asp (128, 173), Phe (148), Tyr (174)
BnGLR57	Ala	Tyr (224)	Lys (197), Val (209), Ala (212), Ile (236)
	Asn	Gln (368)	Met (1, 325), Gly (2, 329), Ile (361), Lys (365)
	Cys	Leu (103)	Ile (43), Met (74), Ala (126), Glu (174), Tyr (307)
	Gln		Pro (612), Arg (845, 848, 854), Gln (849), Val (851), Leu (875), Lys (876)
	Gly	Ala (240)	Leu (213, 243), Met (245), Thr (247), Tyr (250), Trp (252)
	Met		Met (74), Leu (103, 175), Ala (126), Glu (174)
	Ser		Gly (2), Lys (324), Met (325), Ile (361), Gln (368)
BnGLR58	Ala		Phe (578), Trp (583), Val (586), Leu (654), Thr (655), Glu (890), Lys (894)
	Asn	Ser (637)	Ile (597), Glu (601), Thr (618, 635), Met (619), Phe (622), Asp (634), Ala (640)
	Cys	Asp (634), SER (637)	Ile (597), Glu (601), Thr (618, 635), Met (619), Phe (622)
	Gln	Trp (598), Gln (615)	Leu (591), Val (595, 879, 883), Ile (620), Phe (882)
	Gly		Val (586), Leu (627, 651, 654)
	Met	Trp (598)	Leu (591), Ala (594), Val (595, 883), Gln (615), Met (619), Ile (620), Phe (882)
	Ser		Phe (578), Trp (583), Val (586), Leu (627, 654), Glu (890), Lys (894)
BnGLR59	Ala	Ser (407)	Leu (381, 406), Ala (402), Arg (422), Val (426, 541)
	Asn		Val (686, 862), Ile (802), Leu (805, 824)
	Cys		Val (686, 862), Ile (802, 812), Leu (805), Phe (885)
	Gln	Ser (407)	Ala (402), Leu (406, 414), Thr (419), Val (426, 541, 546), Gly (428)
	Gly		Val (317), Leu (350, 361), Ala (360), Ile (384)
	Met	Gln (431)	Thr (291), Met (439), His (454), Gly (520), Gln (521), Val (522), Cys (534)

Continued

	Ser		Leu (406, 414), Ser (407), Ile (539), Val (541, 546)
BnGLR60	Ala	Ala (346), Lys (347)	Leu (118), Phe (120, 356), Gly (345), Leu (348), Val (353), Lys (354)
	Asn	Ala (346), Ile (355)	Phe (120, 356), Gly (345), Lys (347, 354) Leu (348, 350)
	Cys		Leu (118, 348), Phe (120, 356), Gly (345), Ala (346), Lys (347, 354), Val (353)
	Gln		Ser (625), Lys (629), Asn (631, 904), Met (644), Trp (647)
	Gly	Lys (354)	Leu (118, 348), Phe (120), Lys (347), Val (353)
	Met	Ser (625), Lys (629)	Thr (635), Met (644), Asn (904), Pro (906)
	Ser	Lys (347)	Leu (118, 348), Phe (120, 356), Ala (346), Lys (354)
BnGLR61	Ala		Thr (578), Phe (581), Leu (582), Gly (833), Pro (913), Ser (914)
	Asn	Arg (852, 926)	Leu (623), Ile (624), Val (893)
	Cys	Thr (578)	Trp (579), Phe (581), Gly (833), Leu (834), Pro (913), Ser (914)
	Gln		Phe (857), Pro (870), Val (858, 886, 890), Met (863), Ser (867), Leu (871), Ala (872)
	Gly	Asp(6)	Ser(7), Ala (10), Met (298)
	Met	-	Phe (597, 598, 631), Ser (630), Val (916), Arg (926), Gln (927)
	Ser		Val (169, 202), Pro (199), Ile (209, 236), Ala (212)

Table S3. Summary of the predicted post translational modification sites in BnGLR protein sequences

	Phosphorylation sites			N-Glycosylation site	Sumoylation sites	Methylation Sites		
**Proteins**	**Ser**	**Thr**	**Tyr**	**Asn**	**Sumo**	**Lys**	**Arg**	**Glu**
BnGLR1	26	9	6	1	5	1	1	0
BnGLR2	25	9	5	1	5	1	0	0
BnGLR3	25	9	6	1	5	1	0	0
BnGLR4	22	10	5	1	5	1	0	0
BnGLR5	32	12	7	0	5	0	0	0
BnGLR6	31	12	6	0	6	0	1	0
BnGLR7	34	10	12	1	8	1	1	0
BnGLR8	38	7	5	0	8	0	1	0
BnGLR9	35	8	5	0	11	0	0	0
BnGLR10	35	8	7	0	9	0	1	0
BnGLR11	34	8	9	0	8	0	1	0
BnGLR12	34	4	7	1	5	0	1	0
BnGLR13	35	2	5	0	5	0	1	0
BnGLR14	37	8	10	0	7	0	0	0
BnGLR15	33	6	8	0	8	0	0	0
BnGLR16	29	12	6	2	4	0	0	0
BnGLR17	31	10	6	1	5	1	0	0
BnGLR18	30	6	6	1	9	0	1	0
BnGLR19	27	5	7	1	9	0	0	0
BnGLR20	20	9	10	2	13	0	0	0
BnGLR21	21	9	8	1	11	0	4	0
BnGLR22	31	12	8	0	13	1	2	0
BnGLR23	18	6	8	2	6	0	1	0
BnGLR24	38	16	9	1	10	0	1	0
BnGLR25	34	10	7	2	16	0	1	0
BnGLR26	38	10	7	1	15	0	1	0
BnGLR27	33	11	8	1	10	0	1	0
BnGLR28	32	14	8	0	8	0	1	0
BnGLR29	35	12	9	1	3	0	1	0
BnGLR30	24	12	6	1	7	0	1	0
BnGLR31	34	15	8	1	12	0	2	0
BnGLR32	27	13	7	2	7	0	2	0
BnGLR33	32	17	7	0	11	0	2	0
BnGLR34	24	10	11	0	12	1	3	0
BnGLR35	33	16	14	2	10	0	2	0
BnGLR36	28	11	9	0	17	0	1	0
BnGLR37	30	4	13	0	13	0	2	0
BnGLR38	31	4	13	1	13	0	2	0
BnGLR39	31	4	13	1	13	0	2	0

Continued

BnGLR40	44	9	10	1	7	1	4	0
BnGLR41	48	8	10	1	9	1	4	0
BnGLR42	33	13	13	0	6	0	3	0
BnGLR43	34	15	10	0	7	0	5	1
BnGLR44	41	13	10	0	8	0	5	1
BnGLR45	43	11	10	1	8	0	4	1
BnGLR46	43	9	9	0	6	1	4	1
BnGLR47	39	10	9	0	6	1	3	1
BnGLR48	38	9	11	0	6	0	2	1
BnGLR49	42	8	11	0	6	0	3	1
BnGLR50	34	8	11	4	8	0	3	0
BnGLR51	33	8	11	3	7	0	4	0
BnGLR52	33	6	12	4	7	1	3	0
BnGLR53	35	12	11	1	7	1	3	0
BnGLR54	27	13	6	4	7	0	5	0
BnGLR55	26	13	6	4	9	0	5	0
BnGLR56	36	7	7	2	7	0	4	0
BnGLR57	33	7	7	1	9	0	2	0
BnGLR58	40	10	8	1	9	0	2	0
BnGLR59	36	5	7	1	9	0	2	0
BnGLR60	37	5	7	0	5	0	2	0
BnGLR61	34	4	6	0	6	0	2	0
